# Supplementary figures and images for: Immersive NREM2 dreaming preserves subjective sleep depth against declining sleep pressure
Source: PLoS Biol. 2026 Mar 24;24(3):e3003683. doi: 10.1371/journal.pbio.3003683 (PMC13012497; doi:10.1371/journal.pbio.3003683)

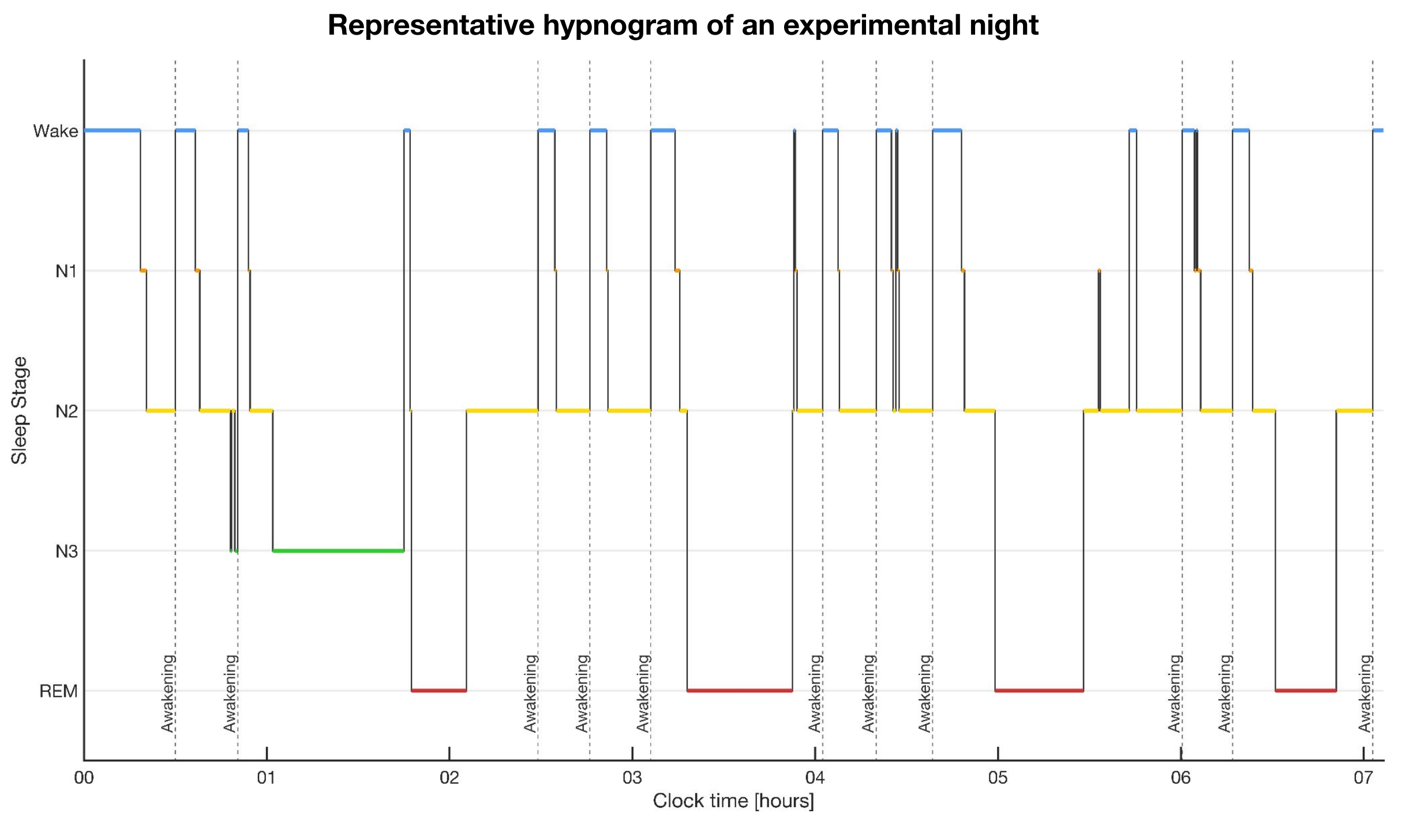

Supplement: S1 Fig — The trace illustrates the progression of sleep stages across the night of a participant in Experiment 1, with the x-axis indicating clock time (00 = midnight) and the y-axis denoting sleep stage (Wake, N1, N2, N3, and REM). Sleep scoring was performed using U-Sleep. Vertical dashed lines mark experimentally induced awakenings delivered during N2 sleep. By restricting experimental interventions to N2, we maximized data collection within a single stage while minimizing potential alterations to the overall sleep architecture and reducing the risk of cross-stage carry-over or interaction effects. (TIF) [file pbio.3003683.s001.tif]

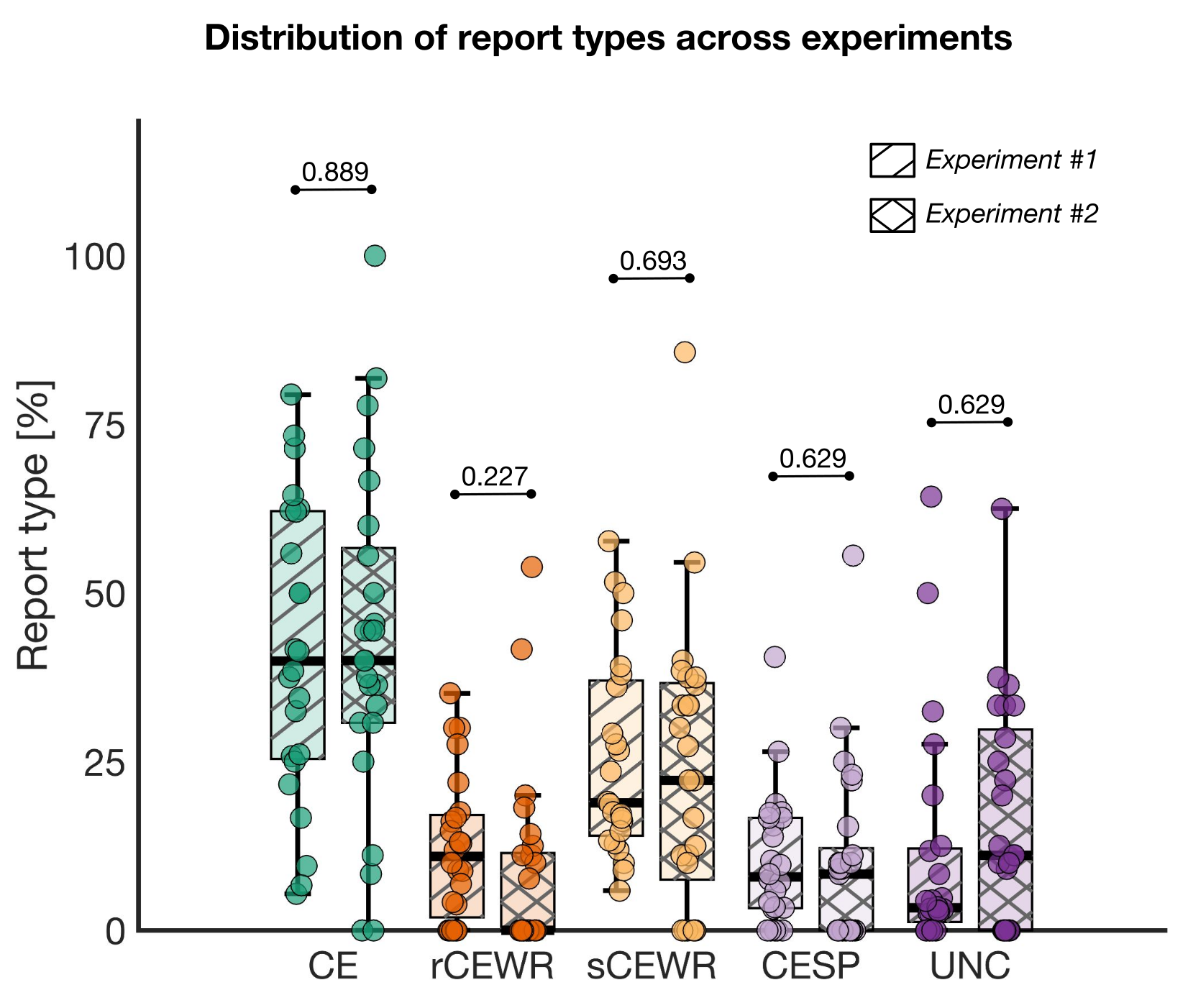

Supplement: S2 Fig — Boxplots show the percentage of each report type for individual participants in Experiment 1 (single oblique line) and Experiment 2 (crossed lines). For each report type, rank-sum tests were used to assess differences between experiments; FDR-corrected q-values are reported above each pair of boxplots. No significant differences were observed across experiments. In box plots, the box spans the interquartile range (IQR), the horizontal line indicates the median, and whiskers extend to the most extreme values within 1.5 × IQR. Report type abbreviations: CE, conscious experience; rCEWR, rich conscious experience without recall of content; sCEWR, simple conscious experience without recall of content; CESP, minimal conscious experience with a sense of presence; UNC, unconsciousness. (TIF) [file pbio.3003683.s002.tif]

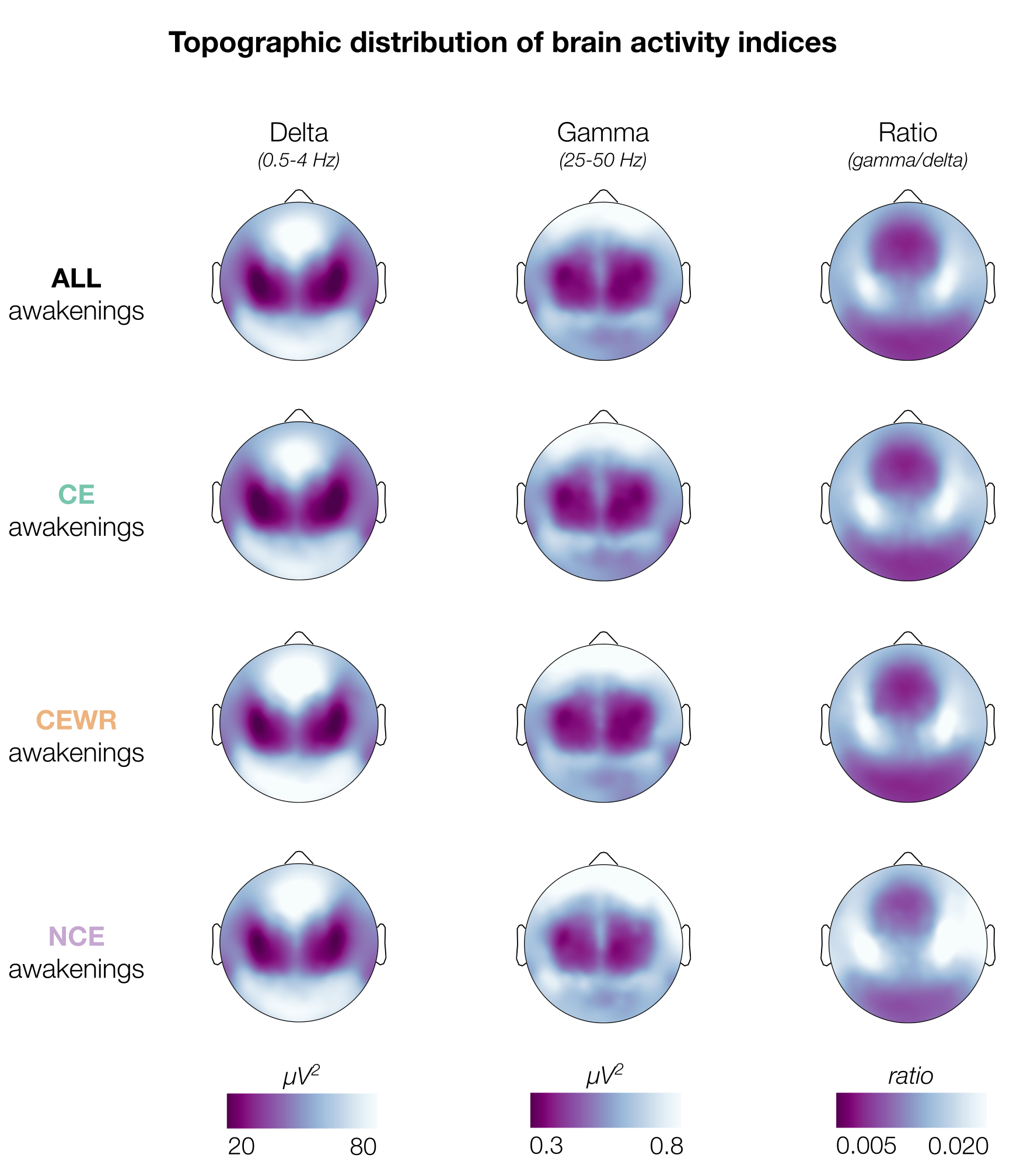

Supplement: S3 Fig — Topographic maps display the average spatial distribution of EEG indices in the 120 s preceding each awakening, separately for all reports, CE reports, CEWR reports, and NCE reports. Values were first averaged across awakenings within participants and then across participants, irrespective of experimental condition. (TIF) [file pbio.3003683.s003.tif]

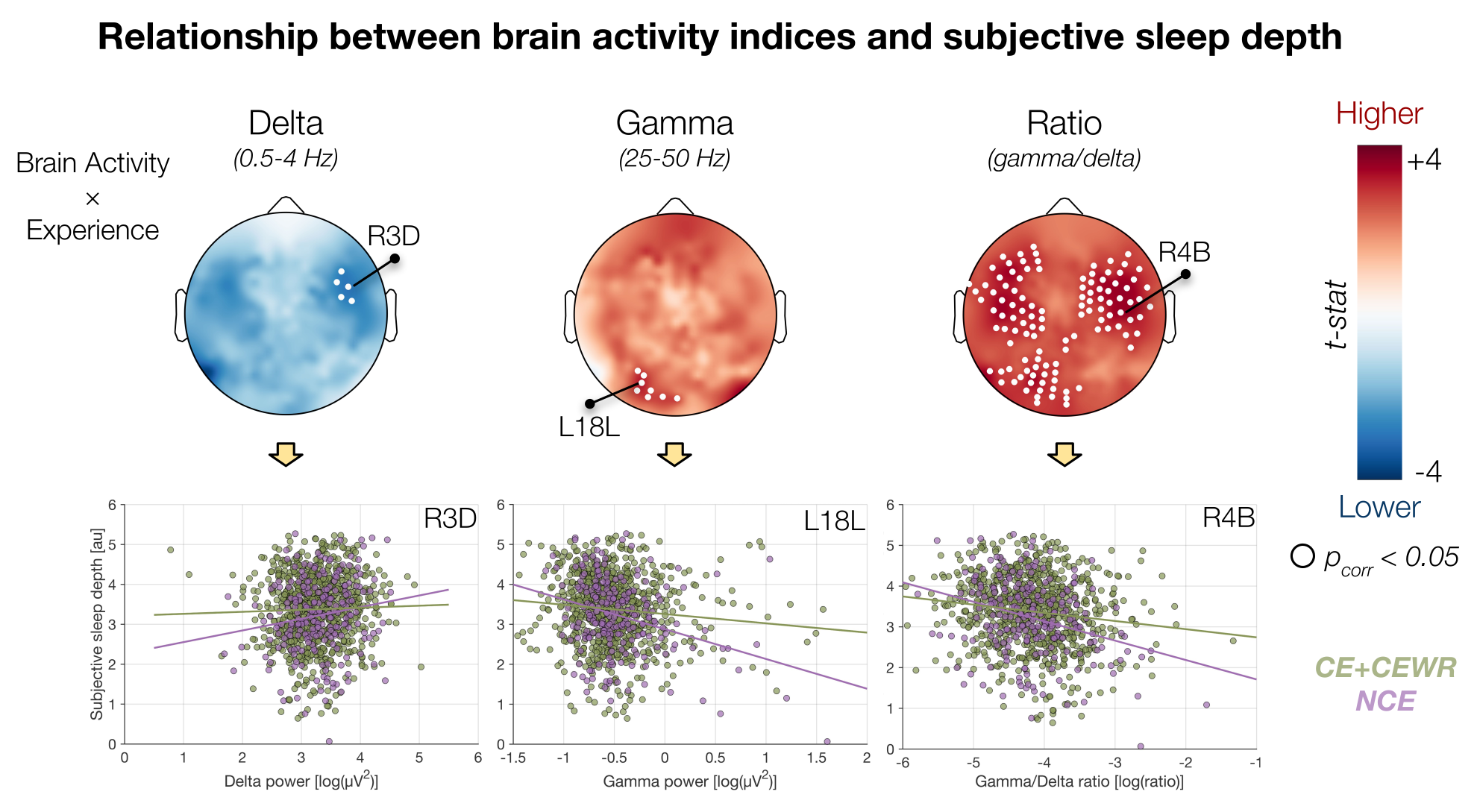

Supplement: S4 Fig — Topographic maps (top) show the interaction between brain activity and conscious experience (CE+CEWR versus NCE) for each brain activity index (the plots are identical to those shown in Fig 2). Wald statistics are projected onto the scalp, with white dots marking significant clusters after cluster-mass correction (corrected p < 0.05). The lower panel displays scatter plots illustrating the relationship between sleep depth and brain activity from a representative electrode (indicated on the topographic maps), shown separately for CE+CEWR (dark green) and NCE (purple). All GLME models included participant as a random intercept and experiment, night, and time of night (relative to midnight) as fixed effects; displayed values are adjusted accordingly. (TIF) [file pbio.3003683.s004.tif]

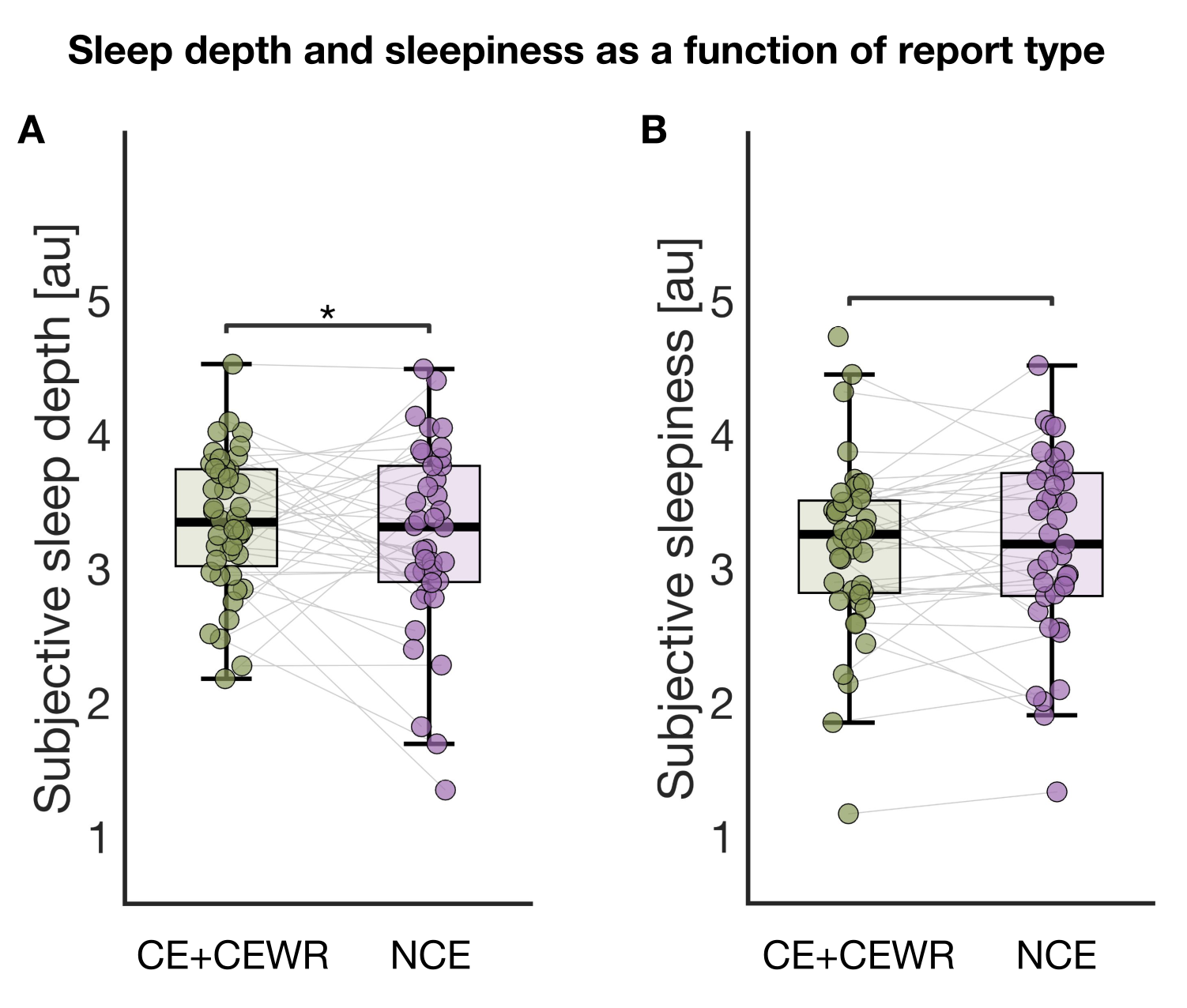

Supplement: S5 Fig — Participants rated sleep depth (A) and sleepiness (B) on a 5-point Likert scale. Each dot represents the average score for an individual participant. Displayed values are adjusted for experiment, night, and time of night. Asterisks indicate statistical significance based on GLME results: *p < 0.05, **p < 0.01, ***p < 0.001. In box plots, the box spans the interquartile range (IQR), the horizontal line indicates the median, and whiskers extend to the most extreme values within 1.5 × IQR. Gray lines link data from the same participant across conditions. Color coding: CE+CEWR (reports of conscious experience) = dark green; NCE (no conscious experience) = purple. (TIF) [file pbio.3003683.s005.tif]

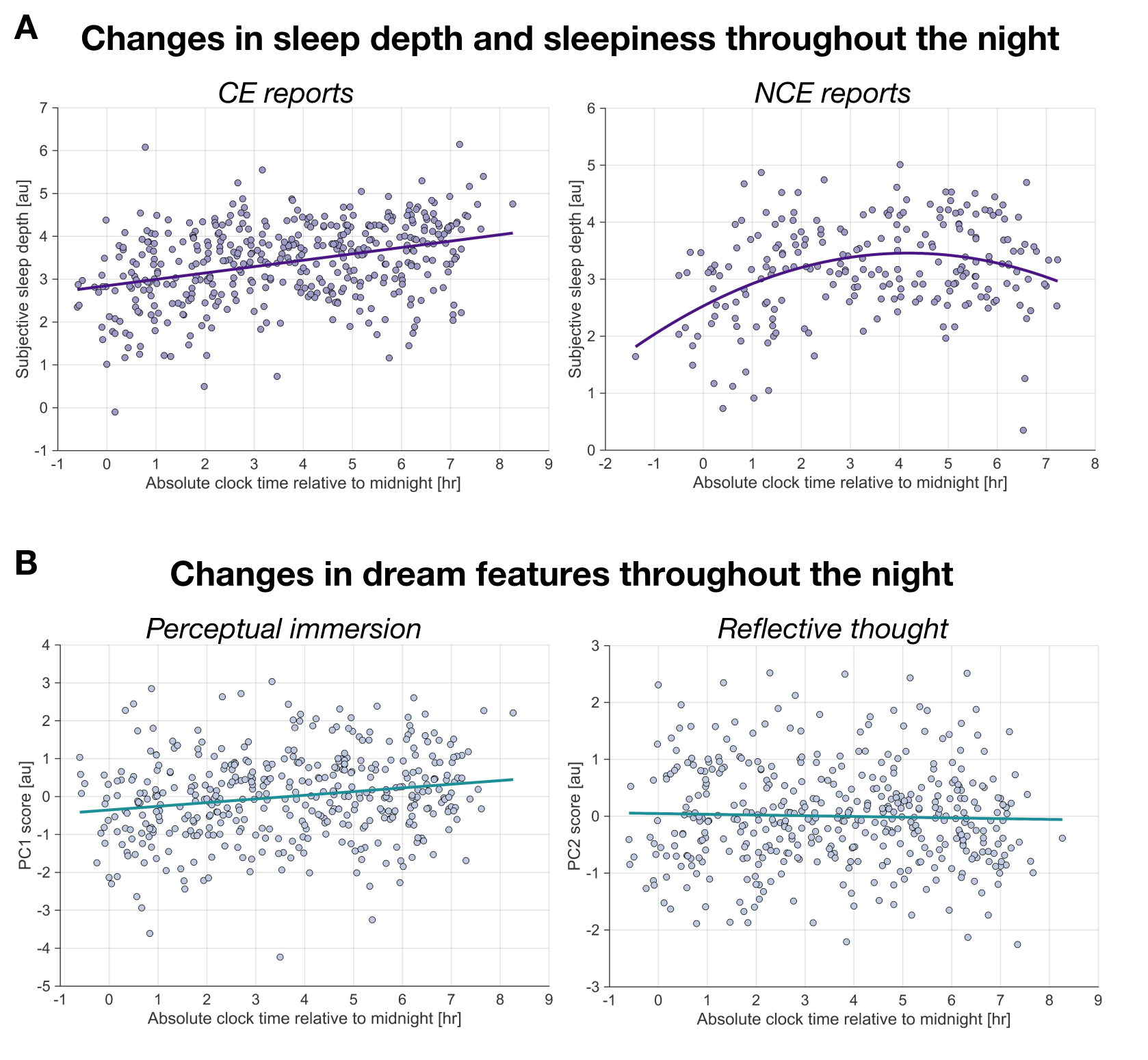

Supplement: S6 Fig — (A) Subjective sleep depth as a function of clock time relative to midnight (0 hour) plotted separately for CE reports (left) and NCE reports (right). (B) Dream perceptual immersion (PC1; left) and reflective thought (PC2; right) components plotted as a function of time of night. All values are adjusted for experiment, night, and participant. In panel A, sleep depth is additionally adjusted for sleepiness scores to account for their shared variance. The left plot in panel B is the same as shown in Fig 5 of the main text, presented here for comparison with the other plots. Each dot represents one observation (N = 432 in panel A, left; N = 228 in panel A, right; N = 427 in panel B). Curves represent the best-fitting polynomial model (linear, quadratic, or cubic) selected using the Bayesian Information Criterion (BIC). (TIF) [file pbio.3003683.s006.tif]
