# Supplementary material for: Immersive NREM2 dreaming preserves subjective sleep depth against declining sleep pressure
Source: PLoS Biol. 2026 Mar 24;24(3):e3003683. doi: 10.1371/journal.pbio.3003683 (PMC13012497; doi:10.1371/journal.pbio.3003683)
Supplement: S1 Table — The table includes summary statistics regarding the participants’ sleep structure (total sleep time and percentages of the different sleep stages) and the performed awakenings (absolute number and percentages of the different report types with respect to the total number of retained awakenings). For the age and the sleep structure indices, values are reported as mean ± standard deviation. (PDF) [file pbio.3003683.s007.pdf]

S1 Table

	Experiment #1	Experiment #2
**Participants** [No.]	24	25
**Sex** [No. females/males]	13/11	12/13
**Age** [years]	26.0 ± 2.3	26.6 ± 4.8
**Total Sleep Time** [min]	330.07 ± 32.80	323.92 ± 32.69
**Stage W** [%]	21.73 ± 7.93	22.07 ± 7.24
**Stage N1** [%]	6.76 ± 3.83	6.36 ± 2.56
**Stage N2** [%]	40.38 ± 7.00	44.47 ± 5.24
**Stage N3** [%]	13.95 ± 4.97	12.35 ± 5.27
**Stage REM** [%]	17.18 ± 4.99	14.76 ± 3.43
**Total Awakenings** [No.]	877	260
**Retained Awakenings** [No. (%)]	783 (89.28%)	241 (92.69%)
**CE reports** [No. (%)]	330 (42.15%)	102 (42.32%)
**CEWR reports** [No. (%)]	284 (36.27%)	80 (33.20%)
**NCE reports** [No. (%)]	169 (21.58%)	59 (24.48%)
