# Supplementary material for: Immersive NREM2 dreaming preserves subjective sleep depth against declining sleep pressure
Source: PLoS Biol. 2026 Mar 24;24(3):e3003683. doi: 10.1371/journal.pbio.3003683 (PMC13012497; doi:10.1371/journal.pbio.3003683)
Supplement: S2 Table — The first two sets of models tested: (1) the main effects of neural predictors (delta power, gamma power, gamma/delta ratio), and (2) interactions between each neural predictor and report type (CE+CEWR versus NCE). The third and fourth sets of models examined the main effects of neural predictors separately for CE+CEWR (3) and NCE (4) reports. For each analysis yielding significant effects, the table presents results from the electrodes with the smallest and largest absolute β coefficients among those that reach significance. All models included experiment, night, and time of night as fixed effects, and participant as a random effect. Reported metrics include the number of observations (N Obs.), adjusted model R² (R² Adj.), likelihood-ratio test p-values (LRT p) comparing full and reduced models excluding the predictor of interest, differences in AIC and BIC (ΔAIC, ΔBIC), estimated regression coefficients (β) with 95% confidence intervals (CI low–high), and corresponding p-values. Positive ΔAIC or ΔBIC values indicate lower AIC/BIC for the full model. (PDF) [file pbio.3003683.s008.pdf]

## S2 Table

| Model | Predictor | Elec. β | N. Obs. | $R^2$ Adj. | LRT p | ΔAIC | ΔBIC | Coeff. β | CI low | CI high | Coeff. p |
|---|---|---|---|---|---|---|---|---|---|---|---|
| **All reports** | Delta | min | 1024 | - | - | - | - | - | - | - | n.s. |
| | | max | 1024 | - | - | - | - | - | - | - | n.s. |
| | Gamma | min | 1024 | 0.231 | 0.00320 | 6.692 | 1.760 | -0.190 | -0.316 | -0.064 | 0.00314 |
| | | max | 1024 | 0.257 | 9.85E-10 | 35.355 | 30.424 | -0.505 | -0.665 | -0.346 | 7.41E-10 |
| | Ratio | min | 1024 | 0.230 | 0.00407 | 6.251 | 1.320 | -0.115 | -0.194 | -0.037 | 0.00408 |
| | | max | 1024 | 0.248 | 2.92E-08 | 28.759 | 23.827 | -0.258 | -0.348 | -0.167 | 2.92E-08 |
| **Interact.** | Delta | min | 1024 | 0.238 | 0.00057 | 10.953 | 1.090 | -0.302 | -0.510 | -0.093 | 0.00457 |
| | | max | 1024 | 0.237 | 0.00033 | 12.059 | 2.196 | -0.336 | -0.550 | -0.122 | 0.00212 |
| | Gamma | min | 1024 | 0.242 | 0.00079 | 10.288 | 0.425 | 0.380 | 0.133 | 0.627 | 0.00260 |
| | | max | 1024 | 0.254 | 0.00028 | 12.379 | 2.516 | 0.467 | 0.195 | 0.739 | 0.00079 |
| | Ratio | min | 1024 | 0.254 | 0.00040 | 11.667 | 1.804 | 0.232 | 0.073 | 0.391 | 0.00431 |
| | | max | 1024 | 0.262 | 0.00008 | 14.908 | 5.045 | 0.333 | 0.146 | 0.519 | 0.00050 |
| **CE + CEWR reports** | Delta | min | 796 | - | - | - | - | - | - | - | n.s. |
| | | max | 796 | - | - | - | - | - | - | - | n.s. |
| | Gamma | min | 796 | 0.259 | 0.00459 | 6.033 | 1.354 | -0.184 | -0.311 | -0.057 | 0.00457 |
| | | max | 796 | 0.279 | 1.80E-07 | 25.242 | 20.562 | -0.425 | -0.583 | -0.267 | 1.82E-07 |
| | Ratio | min | 796 | 0.259 | 0.00437 | 6.124 | 1.445 | -0.135 | -0.228 | -0.043 | 0.00431 |
| | | max | 796 | 0.269 | 0.00006 | 14.113 | 9.434 | -0.208 | -0.309 | -0.107 | 0.00006 |
| **NCE reports** | Delta | min | 228 | - | - | - | - | - | - | - | n.s. |
| | | max | 228 | - | - | - | - | - | - | - | n.s. |
| | Gamma | min | 228 | 0.230 | 0.00231 | 7.289 | 3.860 | -0.357 | -0.586 | -0.129 | 0.00234 |
| | | max | 228 | 0.250 | 0.00001 | 17.243 | 13.813 | -0.723 | -1.038 | -0.409 | 0.00001 |
| | Ratio | min | 228 | 0.228 | 0.00442 | 6.101 | 2.671 | -0.234 | -0.395 | -0.073 | 0.00447 |
| | | max | 228 | 0.260 | 0.00001 | 17.025 | 13.595 | -0.416 | -0.600 | -0.233 | 0.00001 |
