# Supplementary material for: Immersive NREM2 dreaming preserves subjective sleep depth against declining sleep pressure
Source: PLoS Biol. 2026 Mar 24;24(3):e3003683. doi: 10.1371/journal.pbio.3003683 (PMC13012497; doi:10.1371/journal.pbio.3003683)
Supplement: S3 Table — The first two sets of models tested: (1) the main effects of neural predictors (delta power, gamma power, and gamma/delta ratio), and (2) interactions between each neural predictor and report type (CE+CEWR versus NCE). Since no significant interactions were found, we did not examine the main effects of neural predictors separately for CE+CEWR and NCE reports. For each analysis yielding significant effects, the table presents results from the electrodes with the smallest and largest absolute β coefficients among those that reach significance. All models included experiment, night, and time of night as fixed effects, and participant as a random effect. Reported metrics include the number of observations (N Obs.), adjusted model R² (R² Adj.), likelihood-ratio test p-values (LRT p) comparing full and reduced models excluding the predictor of interest, differences in AIC and BIC (ΔAIC, ΔBIC), estimated regression coefficients (β) with 95% confidence intervals (CI low–high), and corresponding p-values. Positive ΔAIC or ΔBIC values indicate lower AIC/BIC for the full model. (PDF) [file pbio.3003683.s009.pdf]

S3 Table

Model	Predictor	Elec. β	N. Obs.	R^2 Adj.	LRT p	ΔAIC	ΔBIC	Coeff. β	CI low	CI high	Coeff. p
**All reports**	Delta	min	1024	-	-	-	-	-	-	-	n.s.
		max	1024	-	-	-	-	-	-	-	n.s.
	Gamma	min	1024	0.443	0.00312	6.734	1.802	-0.148	-0.246	-0.050	0.00311
		max	1024	0.452	0.00001	18.771	13.839	-0.315	-0.449	-0.181	4.60E-06
	Ratio	min	1024	0.443	0.00499	5.883	0.952	-0.091	-0.154	-0.028	0.00491
		max	1024	0.446	0.00023	11.587	6.655	-0.135	-0.207	-0.063	0.00023
**Interact.**	Delta	min	1024	-	-	-	-	-	-	-	n.s.
		max	1024	-	-	-	-	-	-	-	n.s.
	Gamma	min	1024	-	-	-	-	-	-	-	n.s.
		max	1024	-	-	-	-	-	-	-	n.s.
	Ratio	min	1024	-	-	-	-	-	-	-	n.s.
		max	1024	-	-	-	-	-	-	-	n.s.
