# Supplementary material for: Immersive NREM2 dreaming preserves subjective sleep depth against declining sleep pressure
Source: PLoS Biol. 2026 Mar 24;24(3):e3003683. doi: 10.1371/journal.pbio.3003683 (PMC13012497; doi:10.1371/journal.pbio.3003683)
Supplement: S4 Table — Each model compares a specific pair of report types (see ‘Contrast’ column) and includes experiment, night, and time of night as fixed effects, and participant as a random effect. Reported metrics include the number of observations (N Obs.), adjusted model R² (R² Adj.), likelihood-ratio test p-values (LRT p) comparing full and reduced models excluding the predictor of interest, differences in AIC and BIC (ΔAIC, ΔBIC), estimated regression coefficients (β) with 95% confidence intervals (CI low–high), and corresponding p-values. Positive ΔAIC or ΔBIC values indicate lower AIC/BIC for the full model. For comparisons involving the four-level classification (CE, CEWR, CESP, and UNC; Fig 2), false discovery rate (FDR) correction was applied, and the resulting q-value is shown in the final column. Statistically significant effects (q < 0.05) are indicated in bold. (PDF) [file pbio.3003683.s010.pdf]

S4 Table

Contrast	N. Obs.	R^2 Adj.	LRT p	ΔAIC	ΔBIC	Coeff. β	CI low	CI high	Coeff. p	Coeff. q
(CE+CEWR) vs. NCE	1024	0.228	0.01305	4.163	-0.769	-0.182	-0.326	-0.038	**0.01307**	-
CE vs. CEWR	796	0.252	0.86022	-1.969	-6.649	0.012	-0.121	0.144	0.86015	2.1074
CE vs. CESP	538	0.213	1.08E-06	21.785	17.497	-0.552	-0.770	-0.334	**9.10E-07**	**< 0.0001**
CE vs. UNC	554	0.198	0.10778	0.586	-3.731	0.183	-0.040	0.405	0.10705	0.3147
CEWR vs. CESP	470	0.332	1.54E-08	30.002	25.849	0.553	0.365	0.742	**1.50E-08**	**< 0.0001**
CEWR vs. UNC	486	0.267	0.09548	0.779	-3.407	0.169	-0.029	0.368	0.09437	0.3147
CESP vs. UNC	228	0.264	0.00002	15.820	12.391	0.593	0.322	0.865	**0.00003**	**0.0001**
rCEWR vs. sCEWR	361	0.316	0.01176	4.347	0.459	-0.259	-0.459	-0.058	**0.01164**	-
