# Supplementary material for: Immersive NREM2 dreaming preserves subjective sleep depth against declining sleep pressure
Source: PLoS Biol. 2026 Mar 24;24(3):e3003683. doi: 10.1371/journal.pbio.3003683 (PMC13012497; doi:10.1371/journal.pbio.3003683)
Supplement: S5 Table — Each model compares a specific pair of report types (see ‘Contrast’ column) and includes experiment, night, and time of night as fixed effects, and participant as a random effect. Reported metrics include the number of observations (N Obs.), adjusted model R² (R² Adj.), likelihood-ratio test p-values (LRT p) comparing full and reduced models excluding the predictor of interest, differences in AIC and BIC (ΔAIC, ΔBIC), estimated regression coefficients (β) with 95% confidence intervals (CI low–high), and corresponding p-values. Positive ΔAIC or ΔBIC values indicate lower AIC/BIC for the full model. For comparisons involving the four-level classification (CE, CEWR, CESP, and UNC; Fig 2), false discovery rate (FDR) correction was applied, and the resulting q-value is shown in the final column. Statistically significant effects (q < 0.05) are indicated in bold. (PDF) [file pbio.3003683.s011.pdf]

S5 Table

Contrast	N. Obs.	R^2 Adj.	LRT p	ΔAIC	ΔBIC	Coeff. β	CI low	CI high	Coeff. p	Coeff. q
(CE+CEWR) vs. NCE	1024	0.439	0.25960	-0.729	-5.661	0.067	-0.049	0.183	0.25967	-
CE vs. CEWR	796	0.448	0.28339	-0.849	-5.529	0.059	-0.049	0.167	0.28329	0.6941
CE vs. CESP	538	0.302	0.04974	1.850	-2.438	-0.178	-0.355	0.000	0.04979	0.1464
CE vs. UNC	554	0.316	0.00217	7.398	3.081	0.289	0.105	0.472	**0.00207**	**0.0152**
CEWR vs. CESP	470	0.580	0.01721	3.675	-0.478	0.194	0.034	0.353	0.01720	0.0632
CEWR vs. UNC	486	0.593	0.00021	11.761	7.574	0.311	0.147	0.474	**0.00021**	**0.0030**
CESP vs. UNC	228	0.462	0.00738	5.178	1.749	0.330	0.089	0.571	**0.00746**	**0.0366**
rCEWR vs. sCEWR	361	0.620	0.26869	-0.777	-4.665	-0.096	-0.265	0.074	0.26889	-
