# Supplementary material for: Immersive NREM2 dreaming preserves subjective sleep depth against declining sleep pressure
Source: PLoS Biol. 2026 Mar 24;24(3):e3003683. doi: 10.1371/journal.pbio.3003683 (PMC13012497; doi:10.1371/journal.pbio.3003683)
Supplement: S6 Table — All models included experiment, night, and time of night as fixed effects, and participant as a random effect. PC1: perceptual immersion; PC2: reflective thought. Reported metrics include the number of observations (N Obs.), adjusted model R² (R² Adj.), likelihood-ratio test p-values (LRT p) comparing full and reduced models excluding the predictor of interest, differences in AIC and BIC (ΔAIC, ΔBIC), estimated regression coefficients (β) with 95% confidence intervals (CI low–high), and corresponding p-values. Positive ΔAIC or ΔBIC values indicate lower AIC/BIC for the full model. Statistically significant effects (p < 0.05) are indicated in bold. (PDF) [file pbio.3003683.s012.pdf]

S6 Table

Predictor	Elec. β	N. Obs.	R^2 Adj.	LRT p	ΔAIC	ΔBIC	Coeff. β	CI low	CI high	Coeff. p
**Sleep depth**	PC1	427	0.300	3.21E-14	55.602	51.545	0.277	0.208	0.346	**2.96E-14**
	PC2	427	0.244	0.00006	14.074	10.017	-0.195	-0.287	-0.102	**0.00004**
**Sleepiness**	PC1	427	0.252	0.01124	4.427	0.370	0.079	0.018	0.140	**0.01129**
	PC2	427	0.242	0.40896	-1.318	-5.375	-0.032	-0.109	0.044	0.40777
