# Supplementary material for: Immersive NREM2 dreaming preserves subjective sleep depth against declining sleep pressure
Source: PLoS Biol. 2026 Mar 24;24(3):e3003683. doi: 10.1371/journal.pbio.3003683 (PMC13012497; doi:10.1371/journal.pbio.3003683)
Supplement: S7 Table — All models include experiment, night, and time of night as fixed effects, and participant as a random effect. To account for shared variance between subjective sleep depth and sleepiness, each of the models exploring these variables included the other measure as an additional fixed effect. Reported metrics include the number of observations (N Obs.), adjusted model R² (R² Adj.), likelihood-ratio test p-values (LRT p) comparing full and reduced models excluding the predictor of interest, differences in AIC and BIC (ΔAIC, ΔBIC), estimated regression coefficients (β) with 95% confidence intervals (CI low–high), and corresponding p-values. Positive ΔAIC or ΔBIC values indicate lower AIC/BIC for the full model. Statistically significant effects (p < 0.05) are indicated in bold. (PDF) [file pbio.3003683.s013.pdf]

S7 Table

Predicted var.	N. Obs.	R^2 Adj.	LRT p	ΔAIC	ΔBIC	Coeff. β	CI low	CI high	Coeff. p
Seep depth	1024	0.355	0.00E+00	98.943	94.012	0.127	0.103	0.151	**9.81E-24**
Sleepiness	1024	0.533	0.23969	-0.618	-5.549	-0.012	-0.032	0.008	0.23975
Delta power	1024	0.229	9.44E-15	58.016	53.084	-0.072	-0.090	-0.054	**8.53E-15**
Gamma power	1024	0.445	0.20627	-0.403	-5.334	-0.007	-0.018	0.004	0.20634
Gamma/Delta ratio	1024	0.278	1.04E-09	35.249	30.317	0.065	0.044	0.086	**1.02E-09**
PC1	427	0.306	0.00007	13.842	9.785	0.103	0.053	0.154	**0.00006**
PC2	427	0.296	0.50976	-1.565	-5.622	-0.014	-0.054	0.027	0.50956
