# Supplementary material for: Immersive NREM2 dreaming preserves subjective sleep depth against declining sleep pressure
Source: PLoS Biol. 2026 Mar 24;24(3):e3003683. doi: 10.1371/journal.pbio.3003683 (PMC13012497; doi:10.1371/journal.pbio.3003683)
Supplement: S8 Table — Each model included experiment, night, and time of night as fixed effects and participant as a random intercept. PC1: perceptual immersion. Using the U-Sleep automatic staging, we computed the number of minutes separating each N2 awakening from the offset of the closest REM or N3 period within the same sleep bout. To minimize false detections, we excluded REM or N3 fragments containing fewer than three contiguous epochs and tied-rank transformed the distance values to reduce the influence of outliers. Sleep bouts containing no REM or N3 sleep were excluded from analysis. Reported metrics include the number of observations (N Obs.), adjusted model R² (R² Adj.), likelihood-ratio test p-values (LRT p) comparing full and reduced models, differences in AIC and BIC (ΔAIC, ΔBIC), estimated regression coefficients (β) with 95% confidence intervals (CI low–high), and corresponding p-values. Positive ΔAIC or ΔBIC values (i.e., lower AIC/BIC for the full model) indicate that including the stage distance factor improved model fit. No significant effects were identified. (PDF) [file pbio.3003683.s014.pdf]

S8 Table

Stage	Predicted var.	N.Obs.	R^2 Adj.	LRT p	ΔAIC	ΔBIC	Coeff. β	CI low	CI high	Coeff. p
**REM**	Sleep depth	270	0.107	0.84567	-1.962	-5.561	1.4e-04	-0.001	0.002	0.84565
	PC1	118	0.539	0.60904	-1.738	-4.509	0.002	-0.005	0.008	0.60986
**N3**	Sleep depth	252	0.237	0.50273	-1.551	-5.080	5.2e-04	-0.001	0.002	0.50105
	PC1	100	0.486	0.17536	-0.163	-2.769	-0.005	-0.012	0.002	0.17585
