# Supplementary material for: Immersive NREM2 dreaming preserves subjective sleep depth against declining sleep pressure
Source: PLoS Biol. 2026 Mar 24;24(3):e3003683. doi: 10.1371/journal.pbio.3003683 (PMC13012497; doi:10.1371/journal.pbio.3003683)
Supplement: S9 Table — Each model included experiment, night, and time of night as fixed effects and participant as a random intercept. PC1: perceptual immersion. Using the U-Sleep automatic staging, we computed for each N2 awakening the cumulative duration (in minutes) of preceding REM sleep. To minimize false detections, we excluded REM fragments containing fewer than three contiguous epochs, and tied-rank transformed the distance values to reduce the influence of outliers. Analyses were conducted both including all awakenings (top rows) and restricting to awakenings with at least some preceding REM sleep (bottom rows). Reported metrics include the number of observations (N Obs.), adjusted model R² (R² Adj.), likelihood-ratio test p-values (LRT p) comparing full and reduced models, differences in AIC and BIC (ΔAIC, ΔBIC), estimated regression coefficients (β) with 95% confidence intervals (CI low–high), and corresponding p-values. Positive ΔAIC or ΔBIC values (i.e., lower AIC/BIC for the full model) indicate that including the REM amount factor improved model fit. Significant effects (p < 0.05) are shown in bold. (PDF) [file pbio.3003683.s015.pdf]

S9 Table

Data	Predicted var.	N.Obs.	R^2 Adj.	LRT p	ΔAIC	ΔBIC	Coeff. β	CI low	CI high	Coeff. p
**All awakenings**	Sleep depth	1024	0.236	8.2e-05	13.510	8.579	8.2e-04	4.1e-04	0.001	**8.2e-05**
	PC1	427	0.308	0.25084	-0.6814	-4.738	0.001	-8.9e-04	0.003	0.25029
**Awakenings REM > 0**	Sleep depth	697	0.237	0.03237	2.579	-1.968	5.9e-04	5.0e-05	0.001	**0.03237**
	PC1	288	0.429	0.56428	-1.668	-5.331	8.2e-04	-0.002	0.004	0.56387
