# Supplementary material for: Immersive NREM2 dreaming preserves subjective sleep depth against declining sleep pressure
Source: PLoS Biol. 2026 Mar 24;24(3):e3003683. doi: 10.1371/journal.pbio.3003683 (PMC13012497; doi:10.1371/journal.pbio.3003683)
Supplement: S10 Table — Instructions for the volunteers, translated from Italian. (PDF) [file pbio.3003683.s016.pdf]

S10 Table

Prompts	Instructions
1. What was on your mind just before waking up?	We want to know whether you remember having any subjective experience in the moments before the alarm sounded. It is important to note that we are not only referring to classic "dreams," which are typically visual and associated with a story, but to any type of experience, including thoughts, images, sensations, or emotions. If you had no experience, simply report this. Likewise, let us know if you think you had an experience but cannot remember what it was. Depending on your response, you will be asked a follow-up question.
2a. Recall the experience.	If you remember having an experience, please describe it. Focus on the last experience you had in the moments before the alarm sounded. Tell us what you remember. It is okay if you cannot recall all the details or impressions.<br><br>*Example 1*: You were at a party with friends. Suddenly, wolves arrived, and you ran away. You then found yourself in a forest, stopped to look at a plant with very colorful and fragrant flowers, and thought they were beautiful. At that moment, the alarm sounded. The last experience in this case is related to the flowers and includes visual elements (seeing the flowers and their colors), olfactory elements (smelling the flowers), and cognitive elements (thinking that they were beautiful).<br><br>*Example 2*: You were thinking about your to-do list for the next day when you suddenly remembered an urgent deadline, which made you feel anxious. At that moment, the alarm sounded. The last experience in this case includes both the thought about the deadline and the emotional state of anxiety.<br><br>After describing your last experience, you will be asked to estimate how long you had continuous experiences before the alarm sounded. Again, we refer to experiences of any kind, not necessarily forming a single coherent narrative. You do not need to recall specific details of the experience. You should report, approximately, how long you believe you have had continuous experiences before the alarm sounded, not necessarily in one coherent narrative. It is important to note that this question does not refer to the time that passed since the previous awakening but to how much time was occupied by experiences of some kind. For example, if 25 minutes passed since your last awakening but you believe you had subjective experiences only during the last 10 minutes before the current awakening, your response should be "10 minutes."
2b. Do you have the impression that the experience was rich in details or events?	If you had an experience but cannot remember it, you will be asked whether you think it was rich in details or events. Sometimes, even if we cannot recall an experience, we may have the impression that it was vivid, long, or complex. In other cases, we may only have a vague sense of having had an experience without any specific impression of its content. Simply answer "yes" (it was rich in details or events) or "no" (it was not).
2c. Did you have the feeling of being "present"?	If you did not have any experience, you will be asked whether you had a feeling of "presence" before the alarm sounded. By this, we mean a sense of being alive and/or perceiving the present moment and the passage of time. Answer "yes" (I was present) or "no" (I was not).
3. How deeply asleep did you feel before the alarm sounded, on a scale from 1 to 5, where 1 is completely awake and 5 is deeply asleep?	This question refers to your subjective state just before the alarm sounded. Use only whole numbers in your response. "1" means you felt fully alert and awake, "3" indicates an intermediate state between wakefulness and sleep, and "5" means you felt deeply asleep.
4. How sleepy do you feel, on a scale from 1 to 5, where 1 is not sleepy at all and 5 is extremely sleepy?	This question refers to your subjective state after the alarm sounded. Use only whole numbers in your response. "1" means you do not feel sleepy or drowsy at all, "3" indicates moderate sleepiness and drowsiness, and "5" means you feel extremely sleepy and struggle to stay awake.

5. Do you think you perceived a stimulus just before the alarm sounded?	During sleep, it is sometimes possible to perceive (or believe you perceived) events occurring around you. These could be sensory stimuli presented as part of the experiment, unintentional noises from the researchers, or external factors unrelated to the experiment.<br>In some cases, stimuli might be incorporated into the dream experience while still being recognizable as coming from the external environment (e.g., hearing water running from a faucet might be associated with seeing a waterfall in a dream). Please indicate whether you think you consciously perceived any external stimulus just before the alarm sounded. Do not include stimuli that you believe were generated solely by your own mind. If you perceived something, specify whether it was auditory, visual, tactile, or of another kind (e.g., smell, warmth, pain, etc.).
The following questions refer to experiences (dreams, thoughts, images, sensations, emotions) that occurred just before waking up. They do not refer to any previous experiences, even if these were continuous with the ones happening at the moment of the alarm sound.	
6. How vivid was the experience on a scale from 1 to 5, where 1 is not vivid at all and 5 is extremely vivid?	This question refers to the "vividness" of the experience, meaning its level of clarity and sharpness. It is important to note that this question does not only refer to the visual aspect of the experience but to its overall vividness, regardless of its nature, including different types of sensory experiences, thoughts, emotions, or sensations. For example, a very clear and well-defined thought, similar to a waking thought, can be considered a vivid experience. Only whole numbers should be used in the response. A response of "1" means that the experience was unclear and blurry (e.g., a vague and indistinct image), "3" indicates an intermediate level of vividness, while "5" means the experience was extremely sharp and clear (e.g., an image as well-defined as in normal vision).
7. To what extent was the experience perceptual rather than thought-based, on a scale from 1 to 5, where 1 is purely thought and 5 is purely perception?	This question refers to the type of content in the experience. Our experiences can take the form of abstract thoughts (e.g., thinking about things to do the next day) or sensory-perceptual elements (e.g., seeing a landscape, hearing a voice, perceiving a smell, etc.). Some experiences contain both elements (e.g., seeing an object that reminds you of something you need to do the next day). Only whole numbers should be used in the response. A response of "1" means the experience consisted of pure abstract thought without any sensory-perceptual aspects, "3" indicates that thought and perceptual elements were present in equal measure, while "5" means the experience was purely sensory and perceptual, with no thought or reflection.
8. Did the experience contain sensory content? Visual? Auditory? Tactile? Olfactory? Gustatory?	Here, you are asked to specify whether the experience contained at least one recognizable sensory element of a visual, auditory, tactile, olfactory, or gustatory nature. You should respond "yes" or "no" to each type of sensory content mentioned by the experimenter. Keep in mind that it is entirely acceptable to respond "no" to all questions, as in the case of experiences consisting purely of thought, without any sensory or perceptual aspects.
9. To what extent was the experience related to real-life elements, situations, or events encountered during wakefulness, on a scale from 1 to 5, where 1 is not related at all and 5 is fully related?	This question aims to assess how much the experience drew from or referenced memories of elements (e.g., objects, people, places), situations, or events actually encountered during wakefulness. Conscious experiences during sleep can sometimes appear as more or less accurate reworkings of recent (e.g., dreaming about a work meeting that took place that same day or a few days prior) or remote memories (e.g., dreaming about being in a place visited in childhood). Additionally, a single experience may include elements from multiple, seemingly unrelated memories. In other cases, the experience may not reference any specific memory (e.g., dreaming of flying through the clouds). Only whole numbers should be used in the response. A response of "1" means the experience was not directly related to real memories, "3" indicates it was moderately related, while "5" means the experience faithfully reproduced real memories.
10. How bizarre was the experience on a scale from 1 to 5, where 1 is not bizarre at all and 5 is extremely bizarre?	This question refers to the level of "strangeness" in the experience. Experiences can sometimes include elements that seem odd or "unnatural" (e.g., dreaming of flying on the back of an eagle or talking to someone familiar who appears different than usual). It is important to remember that this question asks you to assess how strange or bizarre the experience felt at the moment of waking up, after having lived it. Only whole numbers should be used in the response. A response of "1" means the experience contained no bizarre elements, "3" indicates an intermediate level of bizarreness, while "5" means you found the experience extremely bizarre.

11. To what extent were you aware that you were dreaming, on a scale from 1 to 5, where 1 is not aware at all and 5 is fully aware?	This question refers to your level of awareness that the experience was not real (i.e., it was like a dream). It is important to note that awareness of having a conscious experience during sleep can fluctuate within the experience itself. Sometimes, we temporarily become aware but then forget about it, while other times, we realize it right before waking up. Your response should reflect your level of awareness during the last experience before waking up. Only whole numbers should be used in the response. A response of "1" means there was no awareness of being asleep, "3" indicates a state of uncertainty (e.g., realizing that some aspects of the experience were particularly bizarre but not concluding with certainty that they were unreal), while "5" means there was full awareness.
12. To what extent did you have voluntary control over the content and progression of the experience, on a scale from 1 to 5, where 1 is no control and 5 is full control?	This question refers to your level of voluntary control over the content and flow of the experience. It is important to note that the level of control can vary within the experience itself. Your response should reflect the degree of control you had during the last experience before waking up. Only whole numbers should be used in the response. A response of "1" means there was no ability to voluntarily control the content or direction of the experience, "3" indicates an intermediate level of control (e.g., the ability to make voluntary choices within the experience but not to alter aspects such as characters, settings, etc.), while "5" means there was full control over the experience, including the ability to modify every aspect.
13. How would you rate the emotional valence of the experience on a scale from 1 to 5, where 1 is very negative and 5 is very positive?	This question refers specifically to the valence or "emotional tone" of the experience. Our experiences can be predominantly associated with negative, neutral, or positive emotions. Only whole numbers should be used in the response. A response of "1" means the experience was associated with extremely negative emotions (e.g., a bad dream or nightmare), "3" indicates a neutral emotional content (neither predominantly positive nor predominantly negative), while "5" means the experience was associated with extremely positive emotions. It is important to note that the score does not reflect the strength or intensity of emotions but only the subjective valence as more or less negative/positive, or neutral.
14. How would you rate the emotional intensity of the experience on a scale from 1 to 5, where 1 is very weak and 5 is very strong?	This question specifically refers to the intensity of emotions in the experience. Regardless of their valence, our experiences can be associated with emotional states of varying strength and intensity. Only whole numbers should be used in the response. A response of "1" means the experience was associated with minimal emotional intensity, "3" indicates an intermediate intensity, while "5" means the experience was associated with extremely strong and intense emotions. It is important to remember that the score does not reflect the valence or tone of emotions but only their strength.
15. How confident are you that your memories of the experience are complete and accurate on a scale from 1 to 5, where 1 is not confident at all and 5 is very confident?	This question refers to your level of certainty that what you remember about the experience accurately and completely reflects what actually occurred. Only whole numbers should be used in the response. A response of "1" means you do not feel confident that you remember the experience completely and accurately, "3" indicates an intermediate level of confidence, while "5" means you feel certain that you remember the experience completely and accurately. In other words, a lower score should be given if you believe you have forgotten parts of the experience or are unsure about remembering certain details correctly.
