# Supplementary material for: Immersive NREM2 dreaming preserves subjective sleep depth against declining sleep pressure
Source: PLoS Biol. 2026 Mar 24;24(3):e3003683. doi: 10.1371/journal.pbio.3003683 (PMC13012497; doi:10.1371/journal.pbio.3003683)
Supplement: S11 Table — Each model included experiment (Experiment #1, Experiment #2), night, and time as fixed effects and participant as a random intercept. PC1: perceptual immersion; PC2: reflective thought. Reported metrics include the number of observations (N Obs.), adjusted model R² (R² Adj.), the likelihood-ratio test p-value (LRT p) comparing the full model with a reduced model excluding experiment, the AIC and BIC differences (ΔAIC, ΔBIC), the estimated fixed effect coefficient (β; positive values indicate higher scores in Experiment #2 relative to Experiment #1), its 95% confidence interval (CI low–high), and the coefficient p-value. Positive ΔAIC or ΔBIC values (i.e., lower AIC/BIC for the full model) indicate that including the experiment factor improved model fit. Statistically significant effects (p < 0.05) are shown in bold. (PDF) [file pbio.3003683.s017.pdf]

S11 Table

Predicted var.	N. Obs.	R^2 Adj.	LRT p	ΔAIC	ΔBIC	Coeff. β	CI low	CI high	Coeff. p
Sleep depth	1024	0.224	0.10829	0.579	-4.352	-0.162	-0.359	0.035	0.10642
Sleepiness	1024	0.438	0.00033	10.896	5.965	-0.355	-0.549	-0.1620	**0.00033**
PC1	427	0.306	0.67115	-1.820	-5.877	-0.084	-0.473	0.305	0.67132
PC2	427	0.296	0.67831	-1.828	-5.885	-0.065	-0.374	0.244	0.67823
