# Supplementary material for: Immersive NREM2 dreaming preserves subjective sleep depth against declining sleep pressure
Source: PLoS Biol. 2026 Mar 24;24(3):e3003683. doi: 10.1371/journal.pbio.3003683 (PMC13012497; doi:10.1371/journal.pbio.3003683)
Supplement: S1 Text — (PDF) [file pbio.3003683.s018.pdf]

S1 Text - Additional experimental procedures

MRI data acquisition

All participants underwent an MRI session that included the acquisition of high-resolution anatomical images using a magnetization-prepared rapid gradient echo (MPRAGE) sequence (TR = 7 ms, TE = 3.2 ms, flip angle = 9°, field of view = 180 mm, acquisition matrix = 224 × 224, voxel size = 1 × 1 × 1 mm^3, 180 sagittal slices). Scans were acquired on a Philips 3T Ingenia system equipped with a 32-channel phased-array head coil. MRI data were not included in the present report.

Experiment #1: sensory stimulation before sleep

Participants arrived at the sleep laboratory around 6.30 PM. After EEG cap preparation, they completed a two-hour task session involving either a visual, auditory, or tactile stimulus duration discrimination task or a battery of standardized activities involving all three sensory modalities. Each experimental night was preceded by only one of these tasks, and their order was randomized across participants. Before and after the task session, participants completed three two-minute-long resting state hd-EEG recordings with their eyes open.

In the duration discrimination task, participants were presented with pairs of tones, vibrotactile pulses, or light flashes and asked to judge whether they had identical or different duration. While seated in front of a computer screen, participants maintained fixation on a central black cross throughout the task. To maintain participants' engagement, the duration difference between stimulus pairs was progressively reduced across blocks to increase the difficulty level, with minimum differences set at 400 ms, 200 ms, 100 ms, or 50 ms. Individual stimulus durations ranged from 200 to 800 ms in 50 ms increments, and each pair of stimuli was separated by a 500 ms inter-stimulus interval. Participants responded using designated keys on a numeric keypad. After each given response, a new pair of stimuli was presented. Each difficulty level included two task blocks with 30 stimuli (20% with matched duration). Participants were instructed to respond as fast and as accurately as possible. Feedback on their performance was provided after each stimulus pair by changing the color of the fixation cross to green (correct) or red (wrong) for 500 ms. An additional feedback indicating the percentage of correct responses was shown on the computer screen at the end of each block. Visual stimuli consisted of flashes of the monitor's background obtained by changing the color from gray to white. Auditory stimuli were 1000 Hz tones including five-ms fade-in and fade-out ramps presented through in-hear headphones (*Maxrock*, Guangdong, China). Mechanical vibratory stimuli (80 Hz) were presented using a dedicated stimulator placed on the index finger of the dominant hand (*Tactamp* and *Tactors*, *Dancer Design*, Ingleton, UK).

The standardized activities were selected to engage auditory, visual, and tactile sensory modalities. In the "*guess the sound*" challenge, participants were presented with 50 different

sounds and had to guess their source. Similarly, in the "*guess the soundtrack*" challenge they had to recognize 42 famous movies and series from their soundtrack. Other activities included solving a set of wooden puzzles, building a LEGO dinosaur, completing a "*word search*" game, and completing a large "*connect the dots*" game. The order of the activities was randomized across participants.

Experiment #2: sensory stimulation during sleep

Participants arrived at the sleep laboratory around 8.30 PM. After the EEG cap and the peripheral sensors were mounted, three different stimulation devices were placed on each participant. Specifically, the same vibrotactile stimulator used in Experiment #1 was taped on the index finger of the dominant hand. Moreover, participants were asked to wear in-ear headphones and a modified sleep mask including two red LED lights (*Mallory Sonalert Products Inc.*, IN, US). The headphones were taped to the EEG cap to avoid the risk of them sliding out during the night. The LEDs were positioned bilaterally in 8 mm cutouts in the sleep mask centered above the eyes. They emitted light with a wavelength of 622 nm and an intensity level of 1500 millicandela. These devices were used to deliver single-pulse 50-ms stimulations during the night. Auditory stimuli consisted of 1000 Hz pure tones (including a five-ms ramp-up and five-ms ramp-down) at a stable intensity level of 40 dB. Tactile stimuli were 80 Hz mechanical vibrations, while visual stimuli consisted of a single red light flash. These stimuli were presented in pseudo-random order during N2 sleep with the aim of inducing a K-complex. Awakenings were induced 4 to 6 seconds after stimuli that successfully evoked a K-complex. In addition to stimulation trials, we also performed sham awakenings not preceded by a stimulation. Of note, if a stimulus failed to evoke a K-complex, the stimulation was repeated once more after a minimum of 2 minutes. If a KC was not evoked by the repeated stimulus, a different stimulation modality or a sham awakening were performed after at least 2 minutes to avoid habituation. Thus, sham awakenings were never closely preceded by stimulation. Only sham trials were analyzed in the present study.

Prior to the beginning of the sleep recording, resting-state recordings with eyes closed were acquired in three two-minute-long blocks.
