# Supplementary material for: Immersive NREM2 dreaming preserves subjective sleep depth against declining sleep pressure
Source: PLoS Biol. 2026 Mar 24;24(3):e3003683. doi: 10.1371/journal.pbio.3003683 (PMC13012497; doi:10.1371/journal.pbio.3003683)
Supplement: Attachment — Submitted filename: response_to_reviewers_rev1.docx [file pbio.3003683.s021.docx]

December 17, 2026

We are sincerely grateful to the editor and the anonymous reviewers for their careful evaluation of our manuscript and for the constructive comments and suggestions that have substantially improved the clarity and overall quality of our work. Below, we provide a detailed, point-by-point response to each comment, together with a description of the corresponding changes made to the manuscript. For the convenience of the editor and reviewers, we have included the most relevant parts of the revised text directly within our responses, with modified portions highlighted in red.

In accordance with the reviewers’ suggestions, we have added a new paragraph to the Discussion section of the revised manuscript. This paragraph explicitly addresses the main limitations of our study and discusses their potential implications for future research. Because this addition is relevant to several comments raised by both reviewers, we reproduce the new text below for ease of reference:

Limitations (Discussion section): “*Several limitations of this work, as well as implications for future research, warrant discussion. First, the present study is correlational and therefore cannot definitely establish a causal link between immersive dreaming and perceived sleep depth. Nonetheless, this interpretation aligns with prior evidence linking dreaming to sensory disconnection during sleep* [(Salvesen et al. 2024)](https://www.zotero.org/google-docs/?tAv8cM)*, and with reports associating altered dream phenomenology with poorer subjective sleep quality (e.g.,* [(Stephan et al. 2021)](https://www.zotero.org/google-docs/?7xOXfo)*). Future work should directly test potential causal relationships, for example by manipulating dream occurrence or complexity through controlled sensory-stimulation paradigms. Second, the use of the serial awakening paradigm inevitably fragments sleep and may influence both dream content and perceived sleep depth across the night. This limitation is particularly evident in multi-stage awakening protocols, which typically require numerous awakenings per night and can substantially alter sleep architecture (e.g.,* [(Stephan et al. 2021)](https://www.zotero.org/google-docs/?B3nZ01)*). To mitigate these effects, we deliberately restricted awakenings to N2 sleep, a stage that offers sufficient variability in both physiology and conscious experience, while minimizing disruption of sleep-dependent processes in N3 and REM. Although this approach precluded direct cross-stage comparisons, it enabled an in-depth characterization of neural and experiential fluctuations within a single, well-defined physiological state. Future studies should extend this approach to other sleep stages, particularly N3 and REM, to assess whether the relationships between dream phenomenology, cortical activation, and perceived sleep depth generalize across the sleep cycle. Third, a related concern is the potential for carry-over effects from preceding sleep stages. As in all serial awakening studies, residual physiological or memory traces from prior REM or N3 episodes cannot be completely excluded. To minimize this possibility, participants were explicitly instructed to describe only their final experience immediately before the alarm sound (following procedures from* [(Siclari et al. 2013; 2017; 2018)](https://www.zotero.org/google-docs/?JAbzWp)*). In addition, we conducted supplementary control analyses quantifying the distance between each awakening and the preceding REM or N3 episode. These analyses revealed no significant effects on perceived sleep depth or dream phenomenology (S8 Table; also see S9 Table), suggesting that our findings are unlikely to reflect cross-stage contamination but rather capture intrinsic N2 dynamics*”.

Please note that, during the revision process, we identified four instances in which the time-of-night variable had been incorrectly reported in the database used for analysis. All models were subsequently re-run using the corrected data, and the corresponding tables and figures were updated accordingly. These corrections resulted in only minimal changes to the results. The only notable difference is that the previously significant interaction between experience (CE+CEWR vs. NCE) and *gamma* power in frontal electrodes no longer reached significance at the applied statistical threshold, now appearing as a non-significant trend (Fig. 2A). This minor adjustment does not affect the interpretation of our findings or the overall conclusions of the study.

### **Reviewer #1**

#### 1. This is an ambitious and well-executed study combining high-density EEG with a large number of serial awakenings to investigate how dream phenomenology shapes subjective sleep depth. Michalak and colleagues show that immersive NREM dreams counteract the association between cortical activation and shallower perceived sleep, and propose that immersive dreaming helps sustain the felt depth of sleep across the night, even as physiological sleep pressure wanes. The study potentially makes a strong conceptual contribution to sleep and consciousness research. However, several points require clarification and refinement. Below, I outline major and minor comments to be addressed prior to publication.

We thank the reviewer for the positive assessment of our study. We have carefully addressed all points raised and revised the manuscript to improve clarity, precision, and interpretability. Specific responses to the major and minor comments are provided below.

#### 2. When including the "experience" (exp.) variable in the models, only the interaction with brain activity is reported. It would be important to also report the main effect of experience itself on subjective sleep depth and sleepiness.

We agree that reporting the main effect of *experience*, in addition to its interaction with EEG activity, is important for clarity. However, calculating this main effect directly from the electrode-wise models that include the interaction term would be statistically inappropriate, as the *experience* variable does not vary across electrodes. In those models, experience has identical values for all electrodes and therefore contributes no spatially meaningful information. A more valid and interpretable approach is to test the main effect of *experience* in a separate model that excludes the EEG predictors, which captures its overall influence on subjective sleep depth and sleepiness. These results are already presented in the manuscript in the section “*The relationship between conscious experience and subjective sleep depth*” and summarized in Supplementary Tables S4–S5 (first row). In that analysis, we compared awakenings associated with conscious experiences (CE+CEWR) to those without reported experience (NCE), thus directly testing the main effect of *experience* on both subjective sleep depth and subjective sleepiness. We found that perceived sleep depth was significantly higher following conscious experiences, whereas no significant differences emerged for sleepiness.

To ensure that this is immediately clear to readers, we have now added a cross-reference at the end of the preceding section (where the interaction effects are reported) directing readers to the subsequent section and to Tables S4–S5 for the corresponding main-effect results:

“*This analysis revealed that the relationship between EEG features and subjective sleep depth was significantly influenced by whether a conscious experience occurred (Fig. 2A-B; S4 Fig. and S2 Table; for the main effect of experience also see S4 Table). [...] Importantly, these interaction effects were specific to subjective sleep depth and did not extend to subjective sleepiness (Fig. 2C; S3 Table; also see S5 Table), suggesting a distinct modulation of perceived sleep depth by the presence of ongoing mental activity*”.

#### 3. It would strengthen the modeling approach to include indices of model fit (e.g., AIC, BIC, likelihood-ratio tests) to quantify the variance explained by adding experience or report type variables. This would help assess their explanatory power beyond EEG measures.

We thank the reviewer for this excellent suggestion. In the revised version of our manuscript, we have added indices of model fit for all analyses to quantify the contribution of the main variables of interest. Specifically, each Supplementary Table now reports the results of likelihood-ratio tests (LRTs) comparing the full model with a reduced model excluding the main predictor of interest and the corresponding ΔAIC (*AIC_reduced* - *AIC_full*) and ΔBIC (*BIC_reduced* - *BIC_full*) values between the two models. Positive ΔAIC or ΔBIC values (i.e., lower AIC/BIC for the full model) indicate that including the variable improves model fit. This methodological clarification has also been added to the captions of the Supplementary Tables.

#### 4. Report type was modeled as categorical, which is reasonable, but the categories arguably represent gradations of consciousness that are not equidistant. Did the authors also test an ordinal or continuous representation of consciousness? Even if not ideal, discussing why the categorical approach was chosen would clarify the rationale.

While the report types (CE, CEWR, CESP, NCE) may superficially appear to represent a gradation in the level of consciousness, there is currently no empirical or theoretical basis to treat them as points on a continuous or ordinal scale. On the contrary, both previous literature and current data indicate that at least some of these categories likely capture qualitatively distinct experiential states rather than linearly graded levels of awareness.

In particular, CEWR (*conscious experience without recall of content*) is typically interpreted as reflecting a dream that was experienced but later forgotten, rather than a “weaker” conscious experience. While some authors (e.g., [(Fazekas et al. 2019)](https://www.zotero.org/google-docs/?Z4weUS)) have suggested that dream forgetting may reflect lower vividness or salience, empirical evidence does not fully support a simple monotonic relationship between vividness and recall. For instance, prior studies have shown that CE and CEWR share largely similar neural correlates relative to NCE, differing primarily in *gamma* power, an index possibly related to memory encoding rather than to the level of consciousness per se [(Siclari et al. 2017)](https://www.zotero.org/google-docs/?vKWOIk). Consistently, in our data, although many CEWR reports described simple experiences, approximately one in five referred to rich, immersive dreams that could not be recalled in detail, again suggesting variability in memory encoding/access as a key determinant for this report category. Similarly, relatively simple or low-vividness experiences were also reported among CEs, indicating that content richness and recall ability are only partially overlapping dimensions. Consistent with this interpretation, our analyses revealed that CE and CEWR displayed comparable associations with subjective sleep depth and sleepiness, supporting the view that they represent different memory outcomes of similar conscious states rather than distinct levels of awareness.

The remaining categories also do not map clearly onto a single dimension of consciousness. For instance, CESP (conscious experience with a sense of presence) could represent a level of awareness similar to CE or CEWR but lacking reportable content (similar to mind blanking in wakefulness) or, alternatively, a state of reduced consciousness with minimal experiential content. Thus, the relationship among these categories may be multidimensional, involving at least two partially independent dimensions (one related to the level of awareness and another to the content of experience) making an ordinal representation conceptually problematic.

Given these considerations, treating report type as a categorical variable and comparing categories pairwise provides the most reliable and assumption-free approach. Any attempt to model these data as ordinal or continuous would require strong and currently unsupported assumptions about the relative distances between categories, the directionality of their relationships, and the unidimensionality of consciousness measures; assumptions that are neither theoretically justified nor empirically validated in the current state of the field. We therefore believe that our categorical analysis represents the most conservative and scientifically robust strategy available.

To clarify this rationale for readers, we have added a short note in the Methods section explaining that report type was modeled as a categorical variable to avoid imposing unsupported assumptions about its ordinal structure and to allow for an unbiased examination of potential differences across categories:

“*To investigate the association between subjective sleep depth or sleepiness and report type, separate models were fitted for each pairwise comparison of report categories (e.g., CE vs. NCE; models #5, #6). This approach was chosen to avoid imposing assumptions about an ordinal or continuous relationship among categories, as current evidence does not support a unidimensional or equidistant hierarchy of consciousness levels during sleep*”.

#### 5. The construct of "sleep depth" is ambiguous. Here it is operationalized subjectively, but it is often used physiologically (N2/N3, SWA). Since the dataset is restricted to N2, the generalization to "NREM sleep" more broadly should be tempered, ideally already in the title and abstract.

We thank the reviewer for highlighting this important conceptual ambiguity. In the present study, “sleep depth” refers specifically to the subjective perception of how deeply participants felt they were sleeping, as rated immediately after each awakening. Throughout the manuscript, we consistently use the terms *subjective* or *perceived* sleep depth to avoid confusion with physiological or stage-based definitions. We deliberately focused on NREM2 sleep, as this within-stage approach allowed us to examine electrophysiological and phenomenological variations in perceived sleep depth without confounds arising from the macroscopic differences that separate sleep stages (also see our response to comment #6 of reviewer #2 and the new limitations paragraph at the beginning of this letter).

To ensure clarity and transparency, we have revised the manuscript to make this focus explicit in all relevant sections, including the title and abstract. In particular, the revised title now reads: “*Immersive NREM2 dreaming preserves subjective sleep depth against declining sleep pressure.*”

#### 6. The conclusion that immersive dreams "sustain" perceived sleep depth as sleep pressure wanes is compelling but causal framing is too strong. The data are correlational, and immersive dreams might also preferentially arise in physiologically stable deep states. Please temper this language and acknowledge possible bidirectionality.

We agree with the reviewer that our data are correlational and cannot establish directionality. In the revised manuscript, we have tempered all causal language, replacing formulations such as “dreams sustain” or “preserve” subjective sleep depth with “dreams are associated with” or “may help sustain.” We also explicitly clarify in the Discussion that a causal link cannot be demonstrated in the present study and outline how this question could be addressed in future works.

At the same time, we note that the alternative explanation, that immersive dreams simply emerge in physiologically stable, “deep” states, would imply that both conscious and unconscious N2 periods depend on comparable levels of physiological sleep depth, which seems inconsistent with current evidence. Prior studies indicate that dreaming tends to arise alongside localized increases in wake-like activity, whereas NCE reports are typically associated with more deactivated cortical states. Consistent with this, in our data the positive association between wake-like EEG activity and reduced perceived sleep depth was attenuated when dreams were present. This dissociation argues against a single determinant of physiological and subjective sleep depth.

#### 7. The neural analyses focus mainly on delta, gamma, and their ratio. While justified, this is a relatively coarse view of NREM dynamics. Given the established role of spindles and slow oscillations in sleep depth and mentation, it would be valuable to at least discuss why these features were not included.

We agree that *spindles* (10-16 Hz) and *slow oscillations* (< 1 Hz) are key components of NREM sleep architecture and that their assessment in relation to dreaming and sleep depth would be of great interest. Our analytic strategy was deliberately hypothesis-driven and focused, rather than broadly exploratory, in order to maintain interpretive and statistical rigor. Specifically, the selected frequency bands (*delta*, *gamma*, and their *ratio*) were determined a priori based on strong theoretical and empirical grounds linking these spectral features to both dreaming and subjective sleep depth. In particular, Siclari et al. [(Siclari et al. 2017)](https://www.zotero.org/google-docs/?kdlEa5) demonstrated that dreaming is associated with reduced *delta*, increased *gamma*, and thus a higher *gamma*/*delta* ratio, reflecting localized cortical activation during sleep. Similarly, Lecci et al. [(Lecci et al. 2020, 20)](https://www.zotero.org/google-docs/?FtjCls) and Stephan et al. [(Stephan et al. 2021)](https://www.zotero.org/google-docs/?uHsOAv) reported that subjective sleep depth covaries with the relative balance between high- and low-frequency activity across cortical regions. These findings suggest that the interaction between slow and fast spectral components constitutes a robust and physiologically meaningful index of both dream experiences and perceived sleep depth. Our analyses therefore targeted these same measures to test well-established hypotheses, avoiding an overly exploratory inclusion of additional oscillatory features that could complicate interpretation or inflate multiple-comparison error. In the revised manuscript, we have clarified this rationale in the Results section:

“[...] *Specifically, EEG activity in the two minutes preceding each awakening was analyzed to obtain three frequency-based metrics: low-frequency power (delta: 0.5–4 Hz), high-frequency power (gamma: 25–50 Hz), and the high-to-low-frequency power ratio (gamma/delta), a marker of overall cortical activation* [(Siclari et al. 2017; 2018; Stephan et al. 2021)](https://www.zotero.org/google-docs/?broken=dpQvS5)*. These frequency bands were selected based on prior work linking them to both dreaming and subjective sleep depth. Dreaming has been associated with reduced delta, increased gamma, and a higher gamma/delta ratio, reflecting localized cortical activation during sleep* [(Siclari et al. 2017)](https://www.zotero.org/google-docs/?broken=ev4btx)*. Likewise, subjective sleep depth has been shown to vary with the balance between low- and high-frequency cortical activity* [(Lecci et al. 2020; Stephan et al. 2021)](https://www.zotero.org/google-docs/?broken=iU3pda)”.

Importantly, while *spindles* and *slow oscillations* are functionally significant, particularly in relation to thalamocortical dynamics, their role in regulating sleep mentation and subjective sleep depth remains less well-defined compared to other indices. Moreover, recent evidence highlighted a link between *spindles* and *infraslow oscillations* (~0.02 Hz) associated with noradrenergic modulation and arousability [(Osorio-Forero et al. 2025; Lecci et al. 2017)](https://www.zotero.org/google-docs/?S68xBd). The analysis of these oscillatory patterns requires different analytical frameworks than the broadband spectral approach adopted here. Given these conceptual and methodological considerations we opted to focus on broadband power indices in this work and to examine *spindle* and *infraslow* activity in a dedicated future study.

#### 8. The category of "conscious experience with sense of presence" (CESP) is intriguing but remains conceptually vague and close to simple CEWR. More justification of how this construct was operationalized and why it is distinct from light dream mentation would be useful.

We thank the reviewer for prompting clarification regarding the conceptualization of the CESP category. We respectfully disagree that CESP is conceptually close to (simple) CEWR, as the two categories capture distinct phenomenological conditions: CEWR refers to cases in which individuals report the distinct impression of having dreamt but cannot recall the content (so-called *white dream*), consistent with a lack of memory encoding or access rather than absence of experience, while CESP identifies situations in which individuals report having had no contentful experience, yet acknowledge a minimal awareness of being, aliveness, or the passage of time immediately before awakening. Indeed, CESP was operationalized as cases in which participants explicitly denied recalling any dream content but nonetheless described a contentless awareness of “being there” or “time passing.” These reports indicate a residual form of consciousness without perceptual, narrative, or emotional content, akin to *mind blanking* episodes described during wakefulness [(Andrillon et al. 2021)](https://www.zotero.org/google-docs/?EX883c). In contrast, CEWR represents experiences that were likely contentful but subsequently forgotten. Thus, CEWR reflects contentful consciousness without memory, while CESP reflects contentless consciousness.

We have now expanded the Results section to clarify this operationalization when CESP is first introduced:

“*To test this, we asked participants who reported NCEs to indicate whether they believed they had emerged from a minimal conscious state with a sense of presence (CESP) or from a state of complete unconsciousness (UNC). CESP was defined as a condition in which participants reported a mere awareness of being ‘present’ or of time passing, despite the absence of any specific dream content. Strikingly, only half of NCE reports were classified as UNC (50.15% ± 37.40%), while the remaining half reflected a contentless sense of presence (CESP; sign rank test, p = 0.330).*”

We also added a corresponding clarification in the Methods section specifying how these categories were coded from participants’ verbal and written reports:

“*Finally, for NCE reports, participants were asked whether they had experienced a sense of presence, defined as a feeling of being alive and/or perceiving the present moment or the passage of time prior to awakening (10). Reports affirming this were labeled as conscious experiences with a sense of presence (CESP), while those reporting no such impression were labeled as unconscious (UNC). Thus, CESP identifies cases of minimal, contentless awareness, akin to mind blanking during wakefulness* [(Andrillon et al. 2021)](https://www.zotero.org/google-docs/?CSxq0C)*.*”

#### 9. The two experiments differ substantially, with experiment #2 including stimulation. Although "experiment" is modeled, it would be useful to know whether it had a significant effect on outcomes.

As noted, Experiment #2 included both stimulation and sham awakenings, but only sham awakenings (i.e., those not preceded by any sensory stimulation) were used in the present analyses. Moreover, *experiment* was explicitly modeled as a fixed factor in all GLME models to control for potential differences between the two protocols.

To further evaluate whether the two experimental protocols systematically influenced any of the main outcome measures, we performed an additional series of GLME models for each dependent variable of interest: subjective sleep depth, subjective sleepiness, and the two principal components reflecting dream phenomenology (PC1: *dream immersiveness*, PC2: *reflective thought*). These models included *experiment*, *night*, and *time* of night as fixed effects, and *participant* as a random intercept:

*Y ~ experiment + night + time + (1|participant)*

The results, summarized in Table R1, below, show that the *experiment* factor had no significant effect on subjective sleep depth, dream immersiveness, or reflective thought (all p > 0.1). However, a significant effect emerged for subjective sleepiness, with participants reporting higher sleepiness in Experiment #1 compared to Experiment #2 (p = 0.0003).

This difference most likely reflects minor contextual or procedural variations between the two experiments. For example, participants in Experiment #1 typically completed a task practice session before sleep, which may have contributed to slightly greater overall sleepiness upon awakening. Importantly, this effect was limited to subjective sleepiness and did not extend to subjective sleep depth or dream phenomenology. If anything, this finding further supports our central conclusion that subjective sleep depth and sleepiness are partially dissociable constructs, likely governed by distinct physiological mechanisms.

The results of these analyses are presented in Supplementary Table S11 and are now referenced in the Methods section as follows: “[...] *Of note, we also tested models including time since lights-off instead of absolute clock time, but results were highly consistent with those using absolute time, so only the latter are reported here. The relative influence of the different experimental protocols on the main outcome variables is summarized in Table S11*”.

| **Predicted** | **N.Obs.** | **R^2^ Adj.** | **LRT p** | **ΔAIC** | **ΔBIC** | **Coeff. β** | **CI low** | **CI high** | **Coeff. p** |
| --- | --- | --- | --- | --- | --- | --- | --- | --- | --- |
| Sleep depth | 1024 | 0.224 | 0.10829 | 0.579 | -4.352 | -0.162 | -0.359 | 0.035 | 0.10642 |
| **Sleepiness** | **1024** | **0.438** | **0.00033** | **10.896** | **5.965** | **-0.355** | **-0.549** | **-0.1620** | **0.00033** |
| PC1 | 427 | 0.306 | 0.67115 | -1.820 | -5.877 | -0.084 | -0.473 | 0.305 | 0.67132 |
| PC2 | 427 | 0.296 | 0.67831 | -1.828 | -5.885 | -0.065 | -0.374 | 0.244 | 0.67823 |

*Table R1. Results of the GLME analyses examining the effect of the experimental protocol (Experiment #1 vs. Experiment #2) on subjective sleep depth, subjective sleepiness, and dream phenomenology (PC1: perceptual immersion; PC2: reflective thought). Each model included experiment, night, and time as fixed effects and participant as a random intercept. Reported metrics include the number of observations (N Obs.), adjusted model R² (R² Adj.), the likelihood-ratio test p-value (LRT p) comparing the full model with a reduced model excluding experiment, the AIC and BIC differences (ΔAIC, ΔBIC), the estimated fixed-effect coefficient (β; positive values indicate higher scores in Experiment #2 relative to Experiment #1), its 95% confidence interval (CI low–high), and the coefficient p-value. Positive ΔAIC or ΔBIC values (i.e., lower AIC/BIC for the full model) indicate that including the experiment factor improved model fit. Statistically significant effects (p < 0.05) are shown in bold.*

#### 10. The "Fig." column in Table 1 does not always correspond to the figure numbering in the manuscript. Please check for consistency.

We thank the reviewer for catching this. During internal revisions some figures were renumbered and the “Fig.” column in Table 1 was not fully updated. We have now reconciled all entries so that the table references match the final figure numbering.

#### 11. It was not immediately clear that only significant betas are reported in the main text. An explicit clarification would avoid confusion.

To improve readability, only statistically significant β coefficients were reported in the main text, while complete model outputs (including both significant and non-significant effects) are provided in the Supplementary Tables. We have now added an explicit clarification in the Results section to make this approach transparent:

“*Analyses were conducted using Generalized Linear Mixed-Effects (GLME) models at the electrode level, with cluster-mass correction applied across electrodes (cluster-forming threshold: uncorrected p < 0.005, minimal cluster size: 3 electrodes, number of permutations: 5,000; see Methods). Each model included experiment, night, and time of night (relative to midnight) as fixed effects and participant as a random intercept to account for interindividual variability. For clarity, only significant results are reported in the main text, while full model outputs are provided in the Supporting Information*”.

#### 12. Awakenings were performed at least 15 minutes after REM offset. Could residual REM dreaming beyond this cutoff still influence reports? Please discuss this possibility.

In our study, experimenters aimed to perform awakenings at least 15 minutes after sleep onset and at least 10 minutes after the end of the last REM episode, based on online visual sleep scoring during the night. However, this interval was applied in real time and may not always correspond precisely to the offline scoring verified according to AASM criteria (as reported in the Methods section, a total of 113 awakenings were excluded as they were not confirmed to have occurred from N2 sleep). To examine whether residual REM or N3 physiology could have influenced participants’ reports, we conducted specific analyses that are described in greater detail in our responses to comments #8 and #9 from reviewer #2. These additional analyses showed that: (i) awakenings preceded by a REM (or N3) period represented about one quarter of the dataset, and (ii) the distance from the last REM (or N3) episode, when present, had no significant association with either subjective sleep depth or dream *perceptual immersion* (PC1). While we cannot entirely rule out subtle carry-over effects, these results strongly suggest that our findings are not driven by cross-stage influences or by residual memory traces from preceding REM episodes.

We have now added this clarification to the Discussion section to emphasize that, although serial awakening studies inherently face the challenge of possible carry-over influences from prior sleep stages, our protocol and supplementary control analyses minimize this concern (see the new limitations paragraph).

#### 13. Frequent awakenings may themselves alter dream content or subjective depth perception across the night. While partially controlled statistically, a brief acknowledgment would be appropriate.

We agree with the reviewer that frequent awakenings could, in principle, influence dream content or the subjective perception of sleep depth across the night. An explicit acknowledgment of this limitation was included in the revised Discussion section (see the new limitations paragraph and our response to comment #6 of reviewer #2).

#### 14. The finding that UNC is rated as deep but associated with the highest sleepiness deserves more discussion. Why would unconscious sleep feel deep yet leave participants more sleepy than immersive dreams?

We agree that the apparent dissociation between perceived sleep depth and post-awakening sleepiness warrants further discussion. Based on prior work on the neural correlates of dreaming [(Siclari et al. 2017; 2018)](https://www.zotero.org/google-docs/?lY8fQ9) and on our current findings, we speculate that dreaming corresponds to a relatively more activated cortical state, characterized by increased high-frequency (‘wake-like’) activity compared with dreamless sleep, as supported also by our finding that the typical association between high-frequency activity and shallower perceived depth is attenuated during dreaming. This higher activation may also scale with dream immersiveness. Such an ‘activated’ state could facilitate a smoother transition to wakefulness, thereby reducing sleep inertia and post-awakening sleepiness despite the subjective feeling of deep sleep. In contrast, awakenings from UNC likely occur from a less activated physiological state, perceived as deep but accompanied by higher sleepiness due to stronger sleep inertia and a higher residual drive to return to sleep.

We have expanded the Discussion to include this interpretation and note that the specific neural correlates of unconscious sleep and conscious experience limited to a sense of presence are currently under investigation and will be reported in a dedicated future study:

“*In contrast to sleep depth, subjective sleepiness followed a different pattern: ratings were highest after unconscious sleep and lowest following any form of conscious experience. The increased sleepiness observed after unconscious sleep may reflect stronger post-awakening sleep inertia, a phenomenon associated with reduced cortical activation and higher slow-wave activity* [(Stephan et al. 2025)](https://www.zotero.org/google-docs/?jfSTVB)*. By contrast, dreaming has been linked to more activated cortical states* [(Siclari et al. 2017)](https://www.zotero.org/google-docs/?zQxDpB)*, which could facilitate a smoother transition to wakefulness. Such transient activation may attenuate sleep inertia, leading to lower perceived sleepiness despite the concurrent feeling of deep sleep. This dissociation indicates that sleep depth and sleepiness reflect partially distinct subjective dimensions, shaped by different neural and psychological processes”.*

### **Reviewer #2**

#### 1. This is a carefully designed and timely study that combines high-density EEG with a serial awakening paradigm to probe how subjective sleep depth is shaped by both neural and experiential factors. The central and intriguing finding is that perceived depth is not only associated with cortical deactivation, as indexed by spectral EEG measures, but is also strongly modulated by the quality of ongoing mental activity. In particular, immersive dream experiences sustain or even enhance the feeling of deep sleep, counteracting the expected decline in subjective depth as homeostatic sleep pressure dissipates. This challenges the traditional view that perceived "deep sleep" is defined solely by reduced cortical activity and unconsciousness. From a conceptual perspective, the work opens an interesting discussion: sleep depth may not only be the passive reflection of cortical silence but can also emerge from active brain processes such as immersive dreaming. Such experiences can create a profound sense of detachment and immersion that subjectively "feels deep," even though physiologically they are accompanied by more wake-like activation. This decoupling of cortical state and subjective depth represents a novel contribution and highlights the importance of integrating phenomenological accounts of dreaming with electrophysiological models of sleep. Overall, the study provides strong data and a valuable perspective on the multidimensional nature of sleep depth. It will be of broad interest to sleep and consciousness researchers, and it invites further work on how experiential richness, not just deactivation, shapes the felt quality of sleep.

We sincerely thank the reviewer for their thoughtful and encouraging evaluation. We have carefully considered all of the reviewer’s comments below and have implemented the suggested clarifications and improvements throughout the revised manuscript.

#### 2. On the critical side I believe that the overall messages of the paper are rather simple and for this the paper is overly long and partly highly redundant. I would strongly suggest shortening the message and streamline the main story.

We agree that the manuscript benefits from a more concise and focused presentation. In the revision, we have shortened the final part of the Introduction (see our response to comment #10) and simplified several sections of the Discussion to reduce redundancy and better emphasize the main conceptual message. That said, the manuscript has inevitably become somewhat longer overall, as we incorporated the extended discussions requested by the reviewers. If the reviewer has specific suggestions for additional sections that could be further condensed or simplified, we would be very happy to comply.

#### 3. The 2nd most critical point for me is that only light N2 sleep awakenings were performed yet strong statements are made about for example REM sleep or immersive dreaming episodes. This needs to be elaborated on.

In light of this and similar feedback from reviewer #1, we have clarified throughout the manuscript that our data and conclusions specifically concern NREM2 sleep, and thus refer primarily to dreaming and subjective sleep depth within this stage (also see our response to comment #5 of reviewer #1). Accordingly, we have updated the title and abstract to reflect this scope more precisely and revised the text throughout to ensure consistent terminology. We have also added this point explicitly to the limitations paragraph of the Discussion. That said, we believe it is useful to consider how our findings may relate conceptually to other sleep conditions not directly examined here. In particular, the observation that REM sleep is typically experienced as a relatively deep state despite its high cortical activation is broadly consistent with our results.

#### 4. Can the authors please clarify what is meant by that sentence? "Indeed, perceived sleep depth was lowest during states with a mere sense of presence and highest during immersive dreaming or deep unconsciousness.". I am not sure if "mere sense of presence" is clear at that point to the reader and it means physiologically?

In this context, “mere sense of presence” refers to minimal forms of awareness during sleep, i.e., experiential states in which participants reported only a vague awareness of “being there”, without perceptual or narrative dream content. To clarify this point, we have revised the relevant passage in the abstract as follows:

“*Yet, this relationship weakened in the presence of dreaming, indicating that immersive conscious experiences may counteract the impact of cortical activation on perceived depth. Indeed, perceived sleep depth was lowest during minimal forms of awareness characterized by a mere sense of presence, and highest during immersive dreaming or deep unconsciousness. Across the night, physiological sleep pressure and subjective sleepiness declined, but perceived sleep depth rose alongside increasing dream immersiveness.*”

#### 5. I also wonder if one could make that final statement in the abstract clearer: "These results challenge the view that deep sleep stems solely from reduced brain activity and suggest that immersive dreaming sustains perceived sleep depth as homeostatic pressure wanes." I assume the point here is that "deep sleep" as discussed in the public (but not "deep NREM sleep", what scientists automatically will understand reading that sentence) is not merely a deactivated brain but also could stem from a feeling of high sleep depth due to a "close loop" such as in immersive dreaming right? I am picky here as I believe that the abstract should already be crystal clear on its own without the main text.

We agree that the final sentence of the abstract could be misinterpreted as referring to physiological “deep NREM sleep,” whereas our study specifically concerns subjective sleep depth. We have therefore revised the sentence to make this distinction explicit:

“*These findings challenge the traditional view that the feeling of deep sleep arises solely from reduced brain activity and suggest instead that immersive dreaming may help sustain the subjective experience of deep sleep as physiological sleep pressure wanes*.”

#### 6. Methodologically I do not understand the rationale to perform the serial awakening paradigm "specifically targeting N2 sleep" as a strength here would be to exactly probe perceived sleep depth by awakening in various sleep stages including REM and deep N3 sleep?? Please clarify.

We thank the reviewer for the opportunity to clarify this important point. While awakening participants from multiple sleep stages could, in principle, enable direct comparisons across the entire sleep spectrum, multi-stage serial awakening protocols carry significant methodological drawbacks that can compromise both sleep integrity and data interpretability.

First, repeated awakenings across all stages inevitably fragment sleep and may disrupt stage-specific physiological and cognitive processes. Consistent with this concern, Stephan et al. [(Stephan et al. 2021)](https://www.zotero.org/google-docs/?Kg7Dhr) reported that serial-awakening nights, compared to baseline nights, show marked alterations in sleep architecture, including reduced efficiency, increased wake and N1 periods, and relative increases in N2 and REM sleep (as percentages of total sleep time, which necessarily decreases). The potential consequences of such fragmentation for dream frequency and perceived sleep depth remain unclear. For instance, repeated interruptions of N3 early in the night could plausibly affect perceived sleep depth in later N2 or REM periods.

Second, to achieve sufficient power across different stages, previous studies have typically performed a large number of awakenings per night, spaced approximately every 20 minutes (e.g., [(Siclari et al. 2013)](https://www.zotero.org/google-docs/?vM5WsW)). This high awakening density further amplifies sleep fragmentation and may reduce the stability of both physiological and experiential measures. In contrast, our targeted design was intentionally conservative, averaging fewer than 10 awakenings per night. This approach minimized disruption to sleep continuity while still yielding a greater number of N2 awakenings per participant than previous multi-stage protocols.

Focusing exclusively on N2 sleep was therefore a deliberate methodological choice rather than a constraint. As outlined in the Introduction, N2 offers substantial variability in both cortical activation and conscious experience, ranging from no awareness to vivid dreaming, while maintaining a relatively stable neurophysiological background. Restricting awakenings to N2 allowed us to examine the neural and experiential determinants of perceived sleep depth within a single, well-defined physiological state, reducing confounds related to large inter-stage differences in EEG activity, arousal thresholds, and dream recall likelihood.

We now discuss these considerations explicitly in the revised Discussion (see the new limitations paragraph), framing them both as a general limitation of multi-stage serial-awakening designs and as a rationale for our methodological focus on N2 sleep, which provides an optimal balance between experimental control, participant comfort, and preservation of sleep integrity.

#### 7. Spell out what you mean with a "lower high-to-low frequency power ration" to be 100% clear please. (unfortunately no pages and line numbering is present). In my document this is page 12, last sentence.

The final part of the Introduction, where this sentence originally appeared, has been extensively revised to avoid anticipating the main results and to focus instead on the study’s primary aims and overarching outcomes (also see our response to comment #10). As part of this revision, we removed the sentence concerning the “high-to-low frequency power ratio.”

#### 8. The authors state the interesting finding that across the night "perceived sleep depth increased" also objective measures of sleep pressure decreased which is a crucial finding for their main statement. I here wonder if the authors can compare the duration of REM episodes in that 2nd half of the night, or the successive minutes spent in REM per individual and whether this also predicts how deep people perceive their sleep. One could speculate that more minutes spent in REM already in the first half may lead to less intense dreaming (in the 2nd half) and less perceived sleep depth. That would allow to further understand that proposed association of the authors.

Prompted by this and the following comment, as well as by comment #12 from Reviewer #1, we conducted additional analyses to test whether preceding N3 or REM sleep had any influence on subjective sleep depth and dream phenomenology reported upon N2 awakening. For these analyses, we used automatic sleep staging from the *U-Sleep* algorithm (also see our response to comment #20).

To specifically address the reviewer’s hypothesis, we examined whether the cumulative amount of REM sleep since the beginning of the night predicted subjective sleep depth or dream *perceptual immersion* (PC1). For each awakening, we computed the cumulative duration of REM sleep (in minutes), excluding isolated REM fragments shorter than three contiguous epochs, which likely represented false detections or unstable sleep periods. Because REM duration values exhibited a highly skewed distribution, we applied a tied-rank transformation to reduce the influence of outliers and ensure a robust model fit. The GLME model had the following structure:

*Y ~ REM_amount + experiment + night + time + (1|participant)*

We found that the cumulative amount of REM sleep preceding the awakening was positively associated with subjective sleep depth (β = 0.0008, CI = [0.0004, 0.0012], p = 0.00008), but not with dream *perceptual immersion* (β = 0.0013, CI = [–0.0009, 0.0034], p = 0.25; Table R2). Similar results were obtained when restricting the analysis to awakenings preceded by at least some REM sleep (*REM_amount* > 0).

| **Data** | **Predicted** | **N.Obs.** | **R^2^ Adj.** | **LRT p** | **ΔAIC** | **ΔBIC** | **Coeff. β** | **CI low** | **CI high** | **Coeff. p** |
| --- | --- | --- | --- | --- | --- | --- | --- | --- | --- | --- |
| All | **Sleep depth** | **1024** | **0.236** | **8.2e-05** | **13.510** | **8.579** | **8.2e-04** | **4.1e-04** | **0.001** | **8.2e-05** |
|  | PC1 | 427 | 0.308 | 0.25084 | -0.6814 | -4.738 | 0.001 | -8.9e-04 | 0.003 | 0.25029 |
| REM > 0 | **Sleep depth** | **697** | **0.237** | **0.03237** | **2.579** | **-1.968** | **5.9e-04** | **5.0e-05** | **0.001** | **0.03237** |
|  | PC1 | 288 | 0.429 | 0.56428 | -1.668 | -5.331 | 8.2e-04 | -0.002 | 0.004 | 0.56387 |

*Table R2. Results of the generalized linear mixed-effects (GLME) analyses testing the relationship between cumulative REM sleep and subjective sleep depth or dream phenomenology (PC1: perceptual immersion). Each model included experiment, night, and time of night as fixed effects and participant as a random intercept. Reported metrics include the number of observations (N Obs.), adjusted model R² (R² Adj.), likelihood-ratio test p-values (LRT p) comparing full and reduced models, differences in AIC and BIC (ΔAIC, ΔBIC), estimated regression coefficients (β) with 95% confidence intervals (CI low–high), and corresponding p-values. Positive ΔAIC or ΔBIC values (i.e., lower AIC/BIC for the full model) indicate that including the REM_amount factor improved model fit. Significant effects (p < 0.05) are shown in bold.*

The absence of an association between REM amount (or distance; see comment #9) and dream immersiveness suggests that increases in dream richness across the night cannot be attributed to a carry-over effect from prior REM dreaming. Moreover, this finding does not support a ‘homeostatic regulation’ of dreaming, whereby intense early-night REM episodes would reduce later dream intensity. Instead, the positive association between cumulative REM sleep and subjective sleep depth likely reflects a broader link between global sleep architecture and perceived depth. As REM periods become more frequent and prolonged in the latter part of the night, after homeostatic N3 pressure has dissipated, the occurrence of sustained REM episodes, particularly within the context of a serial-awakening protocol that fragments sleep, may signal more efficient sleep and greater overall stability. Notably, because REM amount predicted perceived sleep depth but not dream immersiveness, the relationship between REM and perceived depth is unlikely to be mediated by dream intensity or vividness.

These additional analyses have been included in the Supplementary Materials (Table S9) and are now explicitly referenced in the Discussion of the revised manuscript (see new limitations paragraph).

#### 9. Figure 1 is interesting but also needs to state that you only use awakenings out of N2 sleep (if I understand correctly). Do the authors argue that due to the fact that REM gets more in the 2nd half of the night N2 awakenings in the vicinity of REM reflect that state? This is by far my biggest criticism until this point as I find it difficult to argue about immersive dreaming (and associating with REM being cortically not that deactivated) but at the same time not even awakening from REM… In that case I would at least like to see the distribution of how many minutes away where the N2 awakening from the REM episodes in the quarters of the night and read the justification early in text that N2 awakenings close to deep N3 sleep or REM sleep supposedly are biased by content or physiological activations there. Alternatively the authors could analyse how many of their 2min analysis windows actually hit REM or N3 and if these reports were distinct in sleep depth quality as compared to such reaching into N2 also before.

All analyses in this study were restricted to awakenings from N2 sleep, as verified both online and offline (see Methods). To make this explicit, we have added a clear statement to the caption of Fig. 1.

We agree that awakenings occurring near transitions from REM or N3 sleep could, in principle, reflect residual physiological or experiential features of those stages. To address this concern, we conducted additional analyses examining the proximity of each N2 awakening to the nearest REM or N3 episode. Using the *U-Sleep* automatic staging, we computed the number of minutes separating each awakening from the offset of the closest REM or N3 period within the same sleep bout. To minimize false detections, we excluded REM or N3 fragments containing fewer than three contiguous epochs and tied-rank transformed the distance values to reduce the influence of outliers. The GLME model was structured as:

*Y ~ stage_distance + experiment + night + time + (1|participant)*

REM episodes were identified in 270 of the 1,024 analyzed awakenings (26.4%), and N3 episodes in 252 (24.6%), with similar proportions observed when restricting the dataset to dream-report awakenings. Thus, the models assessing REM or N3 proximity were based on approximately one quarter of the full dataset.

| **Stage** | **Predicted** | **N.Obs.** | **R^2^ Adj.** | **LRT p** | **ΔAIC** | **ΔBIC** | **Coeff. β** | **CI low** | **CI high** | **Coeff. p** |
| --- | --- | --- | --- | --- | --- | --- | --- | --- | --- | --- |
| REM | Sleep depth | 270 | 0.107 | 0.84567 | -1.962 | -5.561 | 1.4e-04 | -0.001 | 0.002 | 0.84565 |
|  | PC1 | 118 | 0.539 | 0.60904 | -1.738 | -4.509 | 0.002 | -0.005 | 0.008 | 0.60986 |
| N3 | Sleep depth | 252 | 0.237 | 0.50273 | -1.551 | -5.080 | 5.2e-04 | -0.001 | 0.002 | 0.50105 |
|  | PC1 | 100 | 0.486 | 0.17536 | -0.163 | -2.769 | -0.005 | -0.012 | 0.002 | 0.17585 |

*Table R3. Results of the generalized linear mixed-effects (GLME) analyses testing the relationship between REM or N3 offset distance and subjective sleep depth or dream phenomenology (PC1: perceptual immersion). Each model included experiment, night, and time of night as fixed effects and participant as a random intercept. Reported metrics include the number of observations (N Obs.), adjusted model R² (R² Adj.), likelihood-ratio test p-values (LRT p) comparing full and reduced models, differences in AIC and BIC (ΔAIC, ΔBIC), estimated regression coefficients (β) with 95% confidence intervals (CI low–high), and corresponding p-values. Positive ΔAIC or ΔBIC values (i.e., lower AIC/BIC for the full model) indicate that including the stage_distance factor improved model fit. No significant effects were identified.*

These analyses revealed no significant associations between the distance from the last REM or N3 episode and the main outcome variables subjective sleep depth and dream *perceptual immersio*n (all p > 0.17; Table R3). This indicates that the perception of sleep depth and immersive dreaming are not carry-over effects from adjacent REM or N3 periods. The findings provide support to the fact that the observed relationships between cortical activation, dream phenomenology, and perceived sleep depth are not driven by contamination from nearby stages. Nonetheless, we acknowledge that our analyses cannot completely exclude subtler cross-stage influences. We now highlight this point in the Discussion and note that future studies including different sleep stages will be essential to further characterize the relationships observed in N2 sleep (see new limitations paragraph). The analyses have been included in the Supplementary Materials (Table S8).

#### 10. Until the section where I read "Results" as headline I already quite some results. Can this be better separated from the introduction?

We agree that the previous version of the Introduction contained too many details of our results. Following the PLOS Biology’s author guidelines, we have revised the Introduction so that it now concludes with a concise statement of the study’s aim followed by a brief comment on whether that aim was achieved:

“*Building on this dataset, we sought to identify the neural and experiential correlates of subjective sleep depth during N2. Specifically, we examined whether variations in conscious experience, ranging from complete unconsciousness to immersive dreaming, modulate the relationship between cortical activation and the feeling of deep sleep. Our results reveal that subjective sleep depth varies systematically with both neural activity and dream phenomenology, indicating that immersive conscious experiences may help sustain the subjective sense of deep sleep even as physiological sleep pressure declines.*”

#### 11. Reading the % of conscious experiences, no conscious experiences and CE without recall of content I wonder why they do not sum up to 100%. Please clarify here.

We verified all reported percentages and confirmed that they sum to 100% in all cases, except in Fig. 1, where the total was 99.99% due to a rounding mistake concerning awakenings for which the five-level classification was unavailable because of mislabeling at data acquisition (indicated as “N/A” in the figure). This has been corrected in the revised manuscript.

#### 12. Figure 3 is a bit too complex in my view. E.g. do you really need the scatter plots,… Consider to change and to add what the reader should focus on in these graphs.

Because it was unclear whether the comment referred to Fig. 3 (which includes only a single scatter plot) or to Fig. 2, which displays multiple topographic maps and scatter plots and may appear visually dense, we assumed the latter interpretation. Accordingly, we revised Fig. 2 by moving the scatter plots to the Supporting Information (S4 Fig.), allowing the main figure to highlight the central group-level effects and comparisons. In addition, we refined the figure captions to direct readers’ attention to the key findings.

#### 13. What does the sentence "While both dreaming and dreamless sleep are perceived as deeper than minimal experiences, conscious awareness, regardless of its complexity, appears to mitigate feelings of sleepiness upon awakening." mean? I thought with "dreaming" you refer to "conscious awareness" ? Clarify.

We thank the reviewer for pointing out this ambiguity. The sentence was intended to convey that all forms of conscious experience, from awareness limited to a mere sense of presence to richer, contentful dream experiences, are associated with lower levels of sleepiness upon awakening, compared to states of complete unconsciousness. We have revised the wording for clarity:

“*These results highlight that perceived sleep depth and sleepiness follow distinct trajectories across different states of consciousness. While both contentful dreaming and unconscious sleep are perceived as deeper than minimal conscious states limited to a sense of presence, any form of conscious experience, whether contentful or lacking any reportable content, was associated with reduced sleepiness upon awakening as compared to full unconsciousness*”.

#### 14. Rather than using this unnecessary labeling in Figure 3 "Color coding: CE = green; rich CEWR = dark orange; simple CEWR = light orange; CESP = light purple; UNC = dark purple." (it can be inferred from the bar graphs to the right anyways) the reader would benefit to again have the written out descriptions of these states here.

We revised the caption of Fig. 3 to include the full condition names for clarity. In addition, the original caption incorrectly referred to both rich and simple CEWR, although this subdivision is not represented in the figure. This error has been corrected. The revised caption now reads:

“*CE = conscious experience (green), CEWR = conscious experience without recall of content (orange), CESP = conscious rxperience with sense of presence (light purple), and UNC = unconsciousness (dark purple).*”

#### 15. Next the authors talk about "phenomenological features of conscious experiences" but do not mention that this is a PCA performed on the text scripts of written dreams right? Please clarify on what exactly that PCA is performed on.

We thank the reviewer for highlighting this ambiguity. The PCA described in the manuscript was performed on participants’ self-rated phenomenological features of conscious experiences, not on metrics derived from the textual transcripts of dream reports. Specifically, the analysis included ratings of duration (expressed in seconds and log-transformed), vividness (from 1 to 5), bizarreness (1-5), emotional intensity (1-5), perceptual versus thought-like content (1-5), and awareness of the dreaming state (1-5). We have clarified this both in the Methods and in the Results sections, where the PCA is first introduced, to avoid any misunderstanding. The linguistic and semantic analyses of written dream narratives are ongoing and will be reported in a separate publication.

The Results section was revised as follows:

“*To this end, we applied principal component analysis (PCA) to six self-rated experiential dimensions: subjective duration (expressed in seconds and log-transformed), perceptual vs. thought-like content, vividness, bizarreness, emotional intensity, and awareness of the dreaming state (Likert scales from 1 to 5)*”.

The following changes were instead applied to the Methods section:

“*Participants who reported a conscious experience were asked to indicate the subjective duration of the experience (in seconds, minutes, or hours) and to use 5-point Likert scales (1 to 5) to characterize their experience according to the following dimensions: vividness (from low to high), percentual vs. thought-like content (from exclusively thought-like to exclusively perceptual), bizarreness (from low to high), degree of relatedness to events encountered during wakefulness (from absent to strong), emotional valence (from strongly negative to strongly positive), emotional intensity (from low to high), awareness of being into a dream (from no to high), voluntary control over the dream (from no to full)*.

“*In order to obtain a phenomenological characterization of conscious experiences, a principal component analysis (PCA) was applied to six self-reported features: experience duration (log-transformed), bizarreness, vividness, emotional intensity, degree of perceptual rather than thought-like content, and degree of awareness of the dreaming state. Each feature was normalized (z-scored) prior to PCA*”.

#### 16. Reading this interesting results one of course immediately question oneself that the physiological correlate difference may be here. Please add whether eg more high frequencies in frontal areas may explain the negative association of "reflective thought" and perceived sleep depth. (p.18, 2nd paragraph; "Statistical analyses showed that perceptual immersion was positively associated with subjective sleep depth (β = 0.277, CI = [0.208, 0.346], p < 0.00001), whereas reflective thought was negatively associated with sleep depth (β = -0.195, CI = [-0.287, -0.102], p < 0.00001).")

We thank the reviewer for this insightful comment. To address this point, we performed a topographic GLME analysis using PC1 (*perceptual immersion*) and PC2 (*reflective thought*) scores as predicted variables. As predictors, we included EEG signal power (*delta*, *gamma*, or *gamma*/*delta* ratio), together with the same covariates used in our main models (*experiment*, *night*, and *time* of night), and *participant* as a random effect. This analysis followed the same approach used to assess associations between physiological measures and subjective sleep depth or sleepiness (see Methods in the main text). The model structure was as follows:

*Y ~ brain_activity + experiment + night + time + (1|participant)*

After correction for multiple comparisons, no significant effects were observed in any frequency band for either perceptual immersion or reflective thought (see below Figure R1). While null results may appear unexpected, we believe they are consistent with the complex nature of the dream features identified by the PCA. Indeed, the two PCs likely emerge from the interaction of multiple phenomenological dimensions that do not necessarily co-occur to the same extent across dreams. For example, a dream may feel highly immersive because of strong perceptual vividness but relatively modest emotional involvement, whereas another may feel immersive due to intense emotional engagement or bizarreness despite limited perceptual clarity. Previous work has shown that different aspects of dream content are associated with distinct, region-specific activation patterns; for instance, Siclari et al. [(Siclari et al. 2017)](https://www.zotero.org/google-docs/?ExCysF) and Perogamvros et al. [(Perogamvros et al. 2017)](https://www.zotero.org/google-docs/?JhL94m) reported increases in high-frequency activity in different cortical regions depending on the specific dream contents. We speculate that a similar principle applies here: the neural correlates of perceptual immersion and reflective thought are likely to vary depending on the specific configuration of perceptual, cognitive, and emotional components that characterize each dream. As these features interact in different ways across experiences, no single, uniform neural pattern may adequately capture the complexity of these multidimensional phenomena.

*Figure R1. Relationship between brain activity and dream phenomenology. Scalp topographies illustrate the results of GLME models assessing the relationship between EEG activity indices (delta power, gamma power, and gamma/delta ratio) and the two principal components describing dream phenomenology: perceptual immersion (PC1) and reflective thought (PC2). Each EEG index was tested in a separate model including participant as a random intercept and experiment, night, and time of night (relative to midnight) as fixed effects. Displayed values correspond to t-statistics projected on the scalp for each electrode. No statistically significant effects were identified (corrected p < 0.05). Warmer colors indicate stronger positive associations, cooler colors indicate negative associations.*

Given that a detailed exploration of the specific neural correlates of dream contents lies beyond the scope of the present investigation, if the reviewer agrees, we would prefer not to include the present exploratory analyses in the main text, particularly as this peer-review correspondence will be publicly available upon article acceptance. We are currently conducting dedicated analyses to examine these aspects in greater depth, focusing on how different dream components may interact synergistically to give rise to immersive experiences, and hope to include the results in a future publication.

#### 17. The authors repeatedly talk about EEG activity and spectra but should there also add if this is always a grand-average or if specific topographies are here in the focus. Eg. For the association of Gamma and subjective sleepiness one wonders if this holds true for the whole brain or only certain parts of the brain.

We apologize for the lack of clarity regarding this aspect. All of our topographic analyses were performed at the electrode level, using channel-wise GLME models, and we never referred to or used grand-averaged EEG measures. We agree, however, that the text did not always explicitly specify the spatial localization of the significant effects. To address this, we have revised the relevant sections of the Results to indicate more clearly where (i.e., in which scalp regions or electrode clusters) the described effects were observed.

#### 18. Also here I think it is crucial to clearly distinguish subjectively perceived "deep sleep" and "deep NREM sleep" as scientists may read sentences as the following: "Contrary to the longstanding view that deep sleep…"

We fully agree that the term ‘deep sleep’ can be ambiguous, as it is often interpreted physiologically (i.e., N3 sleep) rather than subjectively. Following this and a similar comment from reviewer #1 (comment #5), we carefully reviewed all relevant sections of the manuscript to ensure that ‘subjective sleep depth’ is clearly identified as the construct under investigation and is consistently distinguished from physiological or stage-based definitions of sleep depth. This distinction is central to our work, which precisely aims to highlight that physiological and subjective dimensions of sleep depth can diverge. The sentence noted by the reviewer has been revised accordingly:

“*Contrary to the longstanding view that subjectively deep sleep is defined by unconsciousness and synchronized cortical activity dominated by high-amplitude slow waves, our findings demonstrate that the feeling of deep sleep can also occur alongside increased cortical activation and vivid, immersive dreaming*.”

#### 19. Please rephrase for better readability and clarifying your main point: "wake-like high-frequency activity... is perceived as less disruptive when accompanied by conscious experience."

The sentence was intended to convey that high-frequency, wake-like cortical activity, which is typically associated with lighter sleep, is perceived as less disruptive to the feeling of deep sleep when it co-occurs with conscious experience (e.g., dreaming). To improve clarity, we have rephrased the sentence as follows:

“First, although wake-like high-frequency activity is typically associated with lighter subjective sleep, this relationship is modulated in the presence of conscious experience, so that wake-like cortical activation appears to be less disruptive to the feeling of deep sleep when it occurs alongside dreaming.”

#### 20. Methodologically I wonder how accurate the U-Sleep automatic algorithm is for sleep staging and whether a subset was also manually scored for validation? Please clarify. Awakenings were not dependent on that algorithm but performed online by well trained personnel?

All awakenings were performed online by trained experimenters who visually monitored EEG, EOG, and EMG signals in real time, following criteria derived from the *American Academy of Sleep Medicine* (AASM) scoring rules. All experimenters were trained in AASM-based staging and followed detailed written procedural manuals to ensure consistent application of awakening criteria across all sessions. The same protocol was used in both experiments, and whether awakenings had occurred during the target stage (N2) was later verified offline through visual inspection by an expert scorer (A.M.) according to standard AASM 2.6 rules.

The *U-Sleep* algorithm [(Perslev et al. 2021)](https://www.zotero.org/google-docs/?6IOL5W) was used exclusively for offline sleep staging after data acquisition to derive descriptive sleep architecture measures. The algorithm has been extensively validated, achieving human-level accuracy (global F1 score: 0.79 ± 0.03). To confirm its performance on our dataset, one experimenter (A.M.) manually rescored a subset of seven randomly selected nights, including nights from both Experiment #1 and Experiment #2. The *U-Sleep* and manual staging showed a high level of agreement, with an average F1 score of 0.74 ± 0.07. Macro F1 was calculated as the unweighted average of per-stage F1 scores. Given this overall good agreement and the prior validation of *U-Sleep*, no additional large-scale manual validation was deemed necessary.

We have now clarified in the Methods section that (i) awakenings were based on online AASM-based visual scoring by trained personnel (see our response to comment #21), (ii) offline manual scoring was used to confirm whether awakenings occurred or not from N2, and (iii) *U-Sleep* was used exclusively for offline sleep staging whose accuracy was verified on our dataset:

“*Recordings were segmented into 362-second-long epochs ending two seconds after the onset of the alarm sound. All segments were assessed offline through visual inspection by one trained experimenter (A.M.) to verify whether the awakening occurred from N2 sleep (as defined according to AASM scoring rules).*”

“*Whole-night recordings were off-line sleep scored using the U-Sleep automatic algorithm to obtain general descriptive statistics regarding sleep structure in each night, participant, and experiment (model version 2.0, 30-s epochs) (52). The following channels were used: L3L (roughly corresponding to F3), L3A (C3), L6A (P3), L16Z (O1), R3R (F4), R3A (C4), R6A (P4), R16Z (O2), and bipolar vertical (VEOGR, R1Z) and horizontal (L1G, R1G) EOG. The data were filtered using a low-pass filter at 0.3 Hz and a high-pass filter at 35 Hz, downsampled to 128 Hz, and re-referenced to the contralateral mastoids (L4H, R4H). One experimenter (A.M.) manually rescored a subset of seven randomly selected nights to verify the algorithm’s accuracy, confirming a high concordance between manual and automated scoring (average F1 score: 0.74 ± 0.07). Given this strong agreement and the prior validation of U-Sleep, no further large-scale manual validation was deemed necessary.*”

#### 21. Awakening criteria or instructions for experiment #1 and #2 were completely identical?

The awakening criteria and instructions were completely identical across Experiments #1 and #2. The same experimenters conducted most of the sessions, occasionally assisted by other trained PhD students or postdoctoral researchers who operated under their supervision and followed standardized manuals. Participants received identical written instructions and underwent the same prerecorded questionnaire procedure in both experiments. We have added a clarification in the Methods section to make this explicit:

“*During the experimental nights, sleep staging was performed online based on the standard criteria outlined in the American Academy of Sleep Medicine (AASM) Scoring Manual v. 2.6 (48). All experimenters received training in AASM-based scoring and followed detailed procedural manuals to ensure full consistency across sessions. Awakenings were induced using a 1.5 s computerized alarm sound after at least 15 minutes from sleep onset and 5 minutes of stable N2 sleep. Awakenings in other sleep stages were avoided to minimize possible alterations to sleep structure and sleep-related processes. The awakening criteria and procedures were identical across Experiments #1 and #2. To minimize possible cross-stage contaminations and memory carry-overs, awakenings within 10 minutes from the end of a REM sleep period were also avoided. Experimenters were instructed to perform up to 4 awakenings per sleep cycle, with a target of 8 to 12 awakenings per night. The experimental sessions concluded around 7 AM. Only data from participants who completed all four experimental nights were analyzed*”.

### **References**

[Andrillon, Thomas, Angus Burns, Teigane Mackay, Jennifer Windt, and Naotsugu Tsuchiya. 2021. “Predicting Lapses of Attention with Sleep-like Slow Waves.” *Nature Communications* 12 (1): 3657. https://doi.org/10.1038/s41467-021-23890-7.](https://www.zotero.org/google-docs/?ydAkHb)

[Fazekas, Peter, Georgina Nemeth, and Morten Overgaard. 2019. “White Dreams Are Made of Colours: What Studying Contentless Dreams Can Teach about the Neural Basis of Dreaming and Conscious Experiences.” *Sleep Medicine Reviews* 43 (February): 84–91. https://doi.org/10.1016/j.smrv.2018.10.005.](https://www.zotero.org/google-docs/?ydAkHb)

[Lecci, Sandro, Jacinthe Cataldi, Monica Betta, Giulio Bernardi, Raphaël Heinzer, and Francesca Siclari. 2020. “Electroencephalographic Changes Associated with Subjective Under- and Overestimation of Sleep Duration.” *Sleep* 43 (11): zsaa094. https://doi.org/10.1093/sleep/zsaa094.](https://www.zotero.org/google-docs/?ydAkHb)

[Lecci, Sandro, Laura M. J. Fernandez, Frederik D. Weber, et al. 2017. “Coordinated Infraslow Neural and Cardiac Oscillations Mark Fragility and Offline Periods in Mammalian Sleep.” *Science Advances* 3 (2): e1602026. https://doi.org/10.1126/sciadv.1602026.](https://www.zotero.org/google-docs/?ydAkHb)

[Osorio-Forero, Alejandro, Georgios Foustoukos, Romain Cardis, et al. 2025. “Infraslow Noradrenergic Locus Coeruleus Activity Fluctuations Are Gatekeepers of the NREM–REM Sleep Cycle.” *Nature Neuroscience* 28 (1): 84–96. https://doi.org/10.1038/s41593-024-01822-0.](https://www.zotero.org/google-docs/?ydAkHb)

[Perogamvros, Lampros, Benjamin Baird, Mitja Seibold, Brady Riedner, Melanie Boly, and Giulio Tononi. 2017. “The Phenomenal Contents and Neural Correlates of Spontaneous Thoughts across Wakefulness, NREM Sleep, and REM Sleep.” *Journal of Cognitive Neuroscience* 29 (10): 1766–77. https://doi.org/10.1162/jocn_a_01155.](https://www.zotero.org/google-docs/?ydAkHb)

[Perslev, Mathias, Sune Darkner, Lykke Kempfner, Miki Nikolic, Poul Jørgen Jennum, and Christian Igel. 2021. “U-Sleep: Resilient High-Frequency Sleep Staging.” *Npj Digital Medicine* 4 (1): 72. https://doi.org/10.1038/s41746-021-00440-5.](https://www.zotero.org/google-docs/?ydAkHb)

[Salvesen, Leila, Elena Capriglia, Martin Dresler, and Giulio Bernardi. 2024. “Influencing Dreams through Sensory Stimulation: A Systematic Review.” *Sleep Medicine Reviews* 74: 101908. https://doi.org/10.1016/j.smrv.2024.101908.](https://www.zotero.org/google-docs/?ydAkHb)

[Siclari, Francesca, Benjamin Baird, Lampros Perogamvros, et al. 2017. “The Neural Correlates of Dreaming.” *Nature Neuroscience* 20 (6): 872–78. https://doi.org/10.1038/nn.4545.](https://www.zotero.org/google-docs/?ydAkHb)

[Siclari, Francesca, Giulio Bernardi, Jacinthe Cataldi, and Giulio Tononi. 2018. “Dreaming in NREM Sleep: A High-Density EEG Study of Slow Waves and Spindles.” *The Journal of Neuroscience* 38 (43): 9175–85. https://doi.org/10.1523/JNEUROSCI.0855-18.2018.](https://www.zotero.org/google-docs/?ydAkHb)

[Siclari, Francesca, Joshua J. LaRocque, Bradley R. Postle, and Giulio Tononi. 2013. “Assessing Sleep Consciousness within Subjects Using a Serial Awakening Paradigm.” *Frontiers in Psychology* 4. https://www.frontiersin.org/journals/psychology/articles/10.3389/fpsyg.2013.00542.](https://www.zotero.org/google-docs/?ydAkHb)

[Stephan, Aurélie M., Jacinthe Cataldi, Amrita Singh Virk, and Francesca Siclari. 2025. “Cortical Activity upon Awakening from Sleep Reveals Consistent Spatio-Temporal Gradients across Sleep Stages in Human EEG.” *Current Biology*, ahead of print, July 16. https://doi.org/10.1016/j.cub.2025.06.064.](https://www.zotero.org/google-docs/?ydAkHb)

[Stephan, Aurélie M., Sandro Lecci, Jacinthe Cataldi, and Francesca Siclari. 2021. “Conscious Experiences and High-Density EEG Patterns Predicting Subjective Sleep Depth.” *Current Biology* 31 (24): 5487-5500.e3. https://doi.org/10.1016/j.cub.2021.10.012.](https://www.zotero.org/google-docs/?ydAkHb)
