# Supplementary material for: Immersive NREM2 dreaming preserves subjective sleep depth against declining sleep pressure
Source: PLoS Biol. 2026 Mar 24;24(3):e3003683. doi: 10.1371/journal.pbio.3003683 (PMC13012497; doi:10.1371/journal.pbio.3003683)
Supplement: Attachment — Submitted filename: response_to_reviewers_rev2.docx [file pbio.3003683.s022.docx]

February 9, 2026

We thank the reviewer for their encouraging and constructive comments. Below, we address each comment point by point and describe the corresponding revisions made to the manuscript. We believe that the suggestions raised in this and the previous review rounds have substantially improved the clarity, rigor, and overall quality of the work.

### **Reviewer #2**

#### 1. This is a very good and detailed revision. Yet I question the argument that NREM-2 awakenings were chosen as this is a more homogeneous state… I believe it is also simply methodological easier to conduct the experiment that way and not fully it is still not fully understandable to me given that N3 and specifically REM dreams are discussed in the literature. But it with no doubt is a very appropriate and sensible revision and a very interesting paper for a broad audience. It could be discussed that the argument that "repeated interruptions of N3 early in the night could plausibly affect perceived sleep depth in later N2" is perhaps even a valid argument for "N2 only" awakenings.

We appreciate the reviewer’s thoughtful skepticism regarding our rationale for focusing on N2 sleep. Although restricting awakenings to a single sleep stage may appear methodologically simpler, this is typically not the case in practice. Serial-awakening experiments require continuous monitoring throughout the night regardless of the number of stages sampled, and targeting multiple stages does not substantially increase experimenter workload. By contrast, restricting awakenings to a single stage introduces extended periods of unsampled sleep, thereby placing greater demands on sustained vigilance by the experimenters. For these pragmatic reasons, and given the overall cost of serial awakening paradigms, many laboratories sample across multiple sleep stages to maximize data yield, independent of the specific theoretical question. However, the subjective and phenomenological consequences of repeatedly awakening participants from multiple sleep stages across the night remain poorly characterized. By limiting awakenings to a single stage, we sought to minimize cross-stage interactions and isolate within-stage variability in subjective experience.

In light of these considerations and the reviewer’s suggestion, we further revised the Introduction and Discussion to clarify the rationale for our focus on N2 sleep, particularly with respect to the potential effects of early-night N3 fragmentation on subsequent perceived sleep depth and dream experience later in the night.

Introduction: “*To investigate this complexity, we analyzed a large dataset of 196 overnight high-density EEG recordings (256 electrodes) from 44 healthy adults (mean age = 26.4 ± 3.9 years; 21 female, 23 male), collected using a serial awakening paradigm specifically targeting N2 sleep. This stage was chosen because it represents the largest proportion of total sleep (~50%), is distributed throughout the whole sleep period, and exhibits broad variability in both the presence of dream experiences and perceived sleep depth (9). By focusing on a single sleep stage, we also aimed to avoid fragmenting other sleep stages and thereby altering their associated physiological functions.*”

Discussion: “*Second, the use of the serial awakening paradigm inevitably fragments sleep and may influence both dream content and perceived sleep depth across the night. This limitation is particularly evident in multi-stage awakening protocols, which typically require numerous awakenings per night and can substantially alter sleep architecture (e.g., (8)). Such alterations may directly influence the processes under investigation, including both dream phenomenology and perceived sleep depth. For example, repeated interruptions of N3 early in the night may interfere with the normal dissipation of homeostatic sleep pressure, potentially biasing subjective reports obtained in later sleep periods. Similarly, repeated awakenings from REM sleep may alter REM pressure or affect dream recall and content later in the night. To mitigate these effects, we deliberately restricted awakenings to N2 sleep, a stage that offers sufficient variability in both physiology and conscious experience, while minimizing disruption of sleep-dependent processes in N3 and REM.*”

#### 2. In the same direction I see the answer to the issue: "the established role of spindles and slow oscillations in sleep depth and mentation, it would be valuable to at least discuss why these features were not included." Also here the authors come up with an elegant answer that they just computed frequency band associations hypothesis-driven like previous papers (Siclari et al., 2017); yet that way scientists never could find new associations and intriguing results. I would like to see at least a sentence in the discussion saying that these are microevents that definitely would be worth analyzing in future studies.

We agree with the reviewer that discovery-oriented approaches are essential for advancing the field. The present dataset is highly complex and affords the investigation of many different hypotheses and metrics. In this study, we therefore chose to focus on a specific, literature-grounded set of assumptions and hypotheses to preserve interpretability and avoid combining analyses based on partially distinct frameworks or models. Additional analyses targeting other aspects of sleep depth and dreaming are currently underway and will be addressed in future, dedicated publications.

In response to the reviewer’s suggestion, we have now added an explicit statement in the Discussion acknowledging that sleep microevents, including spindles and K-complexes, fall beyond the scope of the present work but represent an important and promising direction for future studies.

Discussion: “*As with many aspects of sleep physiology, optimal dream expression may lie within a balanced range, with deviations in either direction contributing to poor sleep experiences. Future work should determine whether targeted modulation of dream phenomenology, through behavioral, cognitive, or pharmacological approaches, can enhance sleep quality and help restore the felt depth of sleep in vulnerable populations. Moreover, while the present study delineates distinct subjective states during sleep, future investigations will be needed to further refine their underlying neural characterization. In particular, it will be important to examine how slowly evolving changes in global sleep dynamics interact with faster fluctuations and discrete microevents, such as sleep spindles or K-complexes, to shape moment-to-moment conscious experience and perceived sleep depth*”.

#### 3. Lastly I suggest that Figures like Figure 5 also have the end of scales in the axis. E.g. here "high" and "low" subjective sleep depth (panel A), etc. next to the y-axis (left and right) to be more intuitive to understand.

We thank the reviewer for this helpful suggestion. Accordingly, we have revised Figures 2, 3, 4, and 5 to include descriptive labels at the ends of the relevant axes (e.g., “low” and “high” subjective sleep depth)
